# Maternal probiotic supplementation protects against PBDE-induced developmental, behavior and metabolic reprogramming in a sexually dimorphic manner: Role of gut microbiome

**DOI:** 10.1007/s00204-024-03882-4

**Published:** 2024-11-09

**Authors:** Maximillian E. Denys, Elena V. Kozlova, Rui Liu, Anthony E. Bishay, Elyza A. Do, Varadh Piamthai, Yash V. Korde, Crystal N. Luna, Artha A. Lam, Ansel Hsiao, Margarita Currás-Collazo

**Affiliations:** Department of Molecular Cell and Systems Biology, University of California, Riverside, CA, 92521, USA; Department of Molecular Cell and Systems Biology, University of California, Riverside, CA, 92521, USA; Neuroscience Graduate Program, University of California, Riverside, CA, USA; Department of Microbiology and Plant Pathology, University of California, Riverside, CA, USA; Department of Molecular Cell and Systems Biology, University of California, Riverside, CA, 92521, USA; Department of Microbiology and Plant Pathology, University of California, Riverside, CA, USA; Division of Biomedical Sciences, School of Medicine, University of California Riverside, Riverside, CA, USA; Department of Microbiology and Plant Pathology, University of California, Riverside, CA, USA; Department of Molecular Cell and Systems Biology, University of California, Riverside, CA, 92521, USA; Department of Molecular Cell and Systems Biology, University of California, Riverside, CA, 92521, USA; Department of Molecular Cell and Systems Biology, University of California, Riverside, CA, 92521, USA; Department of Microbiology and Plant Pathology, University of California, Riverside, CA, USA; Department of Molecular Cell and Systems Biology, University of California, Riverside, CA, 92521, USA

**Keywords:** DE-71, Lactobacillus reuteri, Limosilactobacillus reuteri, Body weight, Incisor eruption, Eye opening, Glucose, Tyroid, Endocrine-disrupting chemicals

## Abstract

Polybrominated diphenyl ethers (PBDEs) are endocrine-disrupting persistent organic pollutants (POPs) used as flame retardants in a wide range of commercial applications. We have previously reported neurobehavioral and metabolic reprogramming produced by developmental PBDEs. PBDEs perturb the microbiome, an influencer of life-long health, while probiotic supplementation with *Limosilactobacillus reuteri* (LR) can avert neurobehavioral and endocrine disruption. We, therefore, tested the hypothesis that perinatal maternal LR supplementation would protect gut microbiome richness and diversity, developmental milestones, adult neurobehavior and metabolic homeostasis in PBDE-exposed offspring. C57BL/6N dams were orally exposed to a commercial penta-mixture of PBDEs, DE-71, at 0.1 mg/kg/day, or corn oil vehicle (VEH/CON) during gestation and lactation. Mice offspring received DE-71 or VEH/CON with or without co-administration of LR (ATCC-PTA-6475) indirectly via their mother from gestational day (GD) 0 until postnatal day (P)21 (Cohort 1), or continued to receive LR directly from P22 through adulthood (Cohort 2). Results of fecal 16S rRNA sequencing indicated age- and sex-dependent effects of DE-71 on gut microbial communities. Maternal LR treatment protected against DE-71-induced reduction in α-diversity in P22 females and against β-diversity alterations in P30 males. In females, DE-71 changed the relative abundance of specific bacterial taxa, such as Tenericutes and Cyanobacteria (elevated) and Deferribacterota (reduced). In males, several Firmicutes taxa were elevated, while Proteobacteria, Chlamydiae, and several Bacteroidota taxa were reduced. The number of disrupted taxa normalized by maternal LR supplementation was as follows: 100% in P22 females and 33% in males at P22 and 25% at P30. Maternal LR treatment protected against DE-71-induced delay of postnatal body weight gain in males and ameliorated the abnormal timing of incisor eruption in both sexes. Further, DE-71 produced exaggerated digging in both sexes as well as locomotor hyperactivity in females, effects that were mitigated by maternal LR only in females. Other benefits of LR therapy included normalization of glucose tolerance, insulinto-glucose ratio and plasma leptin in adult DE-71 females (Cohort 2). This study provides evidence that probiotic supplementation can mitigate POP-induced reprogramming of neurodevelopment, adult neurobehavior, and glucose metabolism in association with modified gut microbial community structure in a sex-dependent manner.

## Introduction

Polybrominated diphenyl ethers (PBDEs) are a class of POPs that have been used as ame retardants in a wide range of consumer products since the 1970s. These include household items such as electronics, furniture and textiles as well as construction materials and vehicles (Herbstman et al. 2010). Since PBDEs are added to polymers without forming covalent bonds, they can easily off-gas from products and be released into indoor environments (Zota et al. 2008). Moreover, due to their chemical structure, PBDEs bioaccumulate and persist in the environment for years without significant degradation (Siddiqi et al. 2003). Despite legislative bans on all PBDEs in Europe by 2008, and the voluntary phase-out of penta- and octa-brominated diphenyl ethers (BDE) in the U.S. in 2004, PBDEs continue to pose a significant threat to humans and wildlife. This is further underscored by the EPA’s final rule in 2021, which restricts the use of decabromodiphenyl ether (Carney Almroth et al. 2022; Environmental Protection Agency 2021). Although these restrictions and bans have led to a decline in global trends, PBDEs continue to be released from global waste stocks, existing products, or from recycled materials. As a result, PBDEs are still widely detected in biota and indoor dust world-wide (Zhu et al. 2023). Moreover, the emission of PBDEs is predicted to continue until 2050 due to increased inadvertent recycling rates leading to re-exposure in the general population (Abbasi et al. 2019).

Due to their lipophilic properties, PBDEs bioaccumulate in human tissues, including breast milk and the placenta, resulting in high exposure for fetuses and infants (Marchitti et al. 2017; Ruis et al. 2019; Varshavsky et al. 2020). This, combined with increased hand-to-mouth transfer in toddlers, has resulted in disproportionately greater body burdens in developing infants and children compared to adults (Vuong et al. 2018). Accumulating evidence from human and animal studies has identified that the major health effects associated with PBDE exposures are thyroid endocrine disruption, reproductive and developmental toxicity, and neurotoxicity, especially if exposure occurs during development (Costa and Giordano 2007; Grandjean and Landrigan 2006). These and other reports have raised the concern for potential risks PBDEs pose for neurodevelopmental disorders (NDDs). We have previously demonstrated autistic-like traits in female offspring exposed perinatally to DE-71, a humanly relevant penta-mixture of PBDEs (Kozlova et al. 2022c). In the current study, we investigated the potential use of probiotic treatment to prevent these and other adverse effects produced by developmental PBDE exposure.

Bioremediation efforts have highlighted the potential for deactivating PBDEs in the environment using microbial tools such as anaerobic debromination (*Dehalococcoides* and *Desulfovibrio* spp.) and aerobic debromination of BDE-47 (*Sphingomonas* sp. PH-07 and *Rhodococcus* sp. RR1) (Kim et al. 2007; Robrock et al. 2009). However, using bacteria to avert PBDE bioactivity in vivo has not been examined despite the fact that microbes within the gastrointestinal tract are the first site of xenobiotic metabolism in contact with ingested environmental toxicants (Collins and Patterson 2020; Baralić et al. 2023). Interestingly, certain probiotics such as *Lactobacillus rhamnosus* administered in diet to *Drosophila* can modify the absorption and in vivo toxicity and increase excretion of other POPs such as organophosphate pesticides (Trinder et al. 2016; Baralić et al. 2023). In this way, an undefirstudied possibility is that resident microbiota may mitigate the previously reported neurological and metabolic deficits induced by PBDEs (Kozlova et al. 2020, 2022c). For example, some microbiota such as *Lactobacillus* spp. and *Bifidobacterium* sp., influence the homeostasis of thyroid hormones, which are necessary for normal central nervous system (CNS) development and cognitive function, but that are disrupted by PBDEs (Kodavanti and Curras-Collazo 2010; Fröhlich and Wahl 2019). The probiotic *Limosilactobacillus reuteri* (LR; formerly *Lactobacillus reuteri (*Zheng et al. 2020)), an important resident genus in early life, can improve thyroid gland function, including raising plasma levels of total thyroxine (T4) (Varian et al. 2014, 2017) and improve neurobehavioral outcomes in mice (Buffington et al. 2016). Therefore, we hypothesized that LR may offset neurotoxic effects of early-life exposure to PBDEs.

One of the best established developmental indices that predict adult health is birth body weight. Low birth weight has been associated with an increased risk of chronic diseases and mental health problems in adulthood as well as neurodevelopmental disorders (Lampi et al. 2012; Kim et al. 2024). This relationship is an example of what has been described as the developmental origins of adult health and disease hypothesis (DOHaD) (Barker 1995). This is a concern for unborn children exposed to PBDEs since high maternal plasma levels of BDE-47 in US women are a risk factor for preterm birth (Peltier et al. 2021). Human studies examining the relationship between maternal PBDE body burden and growth measures in their offspring have shown a negative association (Chao et al. 2007; Foster et al. 2011; Robledo et al. 2015; Wu et al. 2010). Consequently, environmental risk factors, including PBDEs, may impact fetal development and adult disease, making them a significant public health concern (Kodavanti et al. 2010; Kozlova et al. 2020). Current knowledge of PBDE effects on other developmental benchmarks is limited (Ta et al. 2011) and it is unknown if *maternal* probiotics can offset their harmful effects. There is mounting evidence suggesting that the gut microbiome plays an essential role in modulating brain development and behavior, in part, via communication between the gut and CNS, known as the gut-brain axis (Heijtz et al. 2011; Mayer et al. 2014). Modi cation of the gut microbiome with probiotics is preferred to be administered indirectly via the mother rather than directly to developing organisms due to the offspring’s immature immune systems.

We and others have shown that early-life exposure to PBDEs is associated with metabolic reprogramming in adult rodents (McIntyre et al. 2015; Wang et al. 2018), suggesting that developmental exposure may contribute to an increased risk for metabolic syndrome and diabetes in adult humans (Lim et al. 2008; Helaleh et al. 2018; Zhang et al. 2016;). In further support of the DOHaD hypothesis, our team demonstrated the diabetogenic effects of perinatal DE-71 (0.1 mg/kg/d), including insulin resistance in female offspring, as well as incomplete glucose clearance, glucose intolerance and altered lipid homeostasis in both sexes (Kozlova et al. 2020, 2022a, 2023). Previous studies have examined the effects of *L. reuteri* 6575 on protection against obesity in mice (Poutahidis et al. 2013). However, potential LR-mediated protection against toxicant-induced glucose dysregulation has not been examined.

The objective of the current study was to investigate the effects of perinatal PBDEs on offspring developmental milestones, adult neurobehavior, metabolic homeostasis and gut dysbiosis. Additionally, we also tested the hypothesis that concomitant maternal supplementation with LR would protect offspring from the adverse health deficits of PBDEs and that this protection would be correlated with improved gut microbial community structure. Mouse dams wereflexposed to DE-71 during pregnancy and lactation, with or without concurrent LR probiotic therapy, and offspring were examined on postnatal days (P)2–30 and through adulthood. We discovered that LR supplementation provided sex-specific protection against DE-71-induced effects, improving birth weight in males and lessening hyperactivity, repetitive behavior and diabetogenic indices in females. These findings indicate that maternal probiotic therapy is beneficial for preventing certain aspects of CNS toxicity in their offspring produced by developmental exposure to PBDEs. A preliminary version of a portion of these findings appears in Kozlova et al. (2022b).

## Materials and methods

### Animal care and maintenance

C57BL/6N mice were obtained from Charles River Laboratories (Raleigh, NC, USA). Mice were group housed 2–4 per cage on corn cob bedding and maintained in a specific pathogen-free vivarium on a 12 h light/dark cycle. The ambient temperature ranged from 20.6 to 23.9 °C and relative humidity was within the limits of 20–70%. Mice were provided rodent chow (Laboratory Rodent Diet 5001; LabDiet, Quakertown, PA, USA) and municipal tap water ad libitum. Procedures on the care and treatment of animals were performed in compliance with the National Institutes of Health *Guide for the Care and Use of Laboratory Animals* and approved by the University of California, Riverside, Institutional Animal Care and Use Committee under protocols 5, 20200018 and 20210031.

### DE-71 dosing solutions

Dosing solutions were prepared as described previously (Kozlova et al. 2020, 2022c). In brief, pentabromodiphenyl ether mixture (DE-71; Lot no. 1550OI18A; CAS 32534-81-9), was obtained from Great Lakes Chemical Corporation (West Lafayette, IN, USA). The DE-71 stock was dissolved in corn oil (Mazola; Summit, IL, USA) and sonicated for 30 min to yifield a low dose: 0.1 mg/kg bw per day (2 μL/g body weight). Vehicle control solution (VEH/CON) contained corn oil only. PBDE doses were chosen to yifield desired body burdens that are relevant to humans (Costa and Giordano 2007) based on the resulting levels and composition of PBDE congeners measured in brain tissue of female (Kozlova et al. 2022c) and male offspring (unpublished observations).

### Developmental exposure to DE-71

**Fig. 1:**
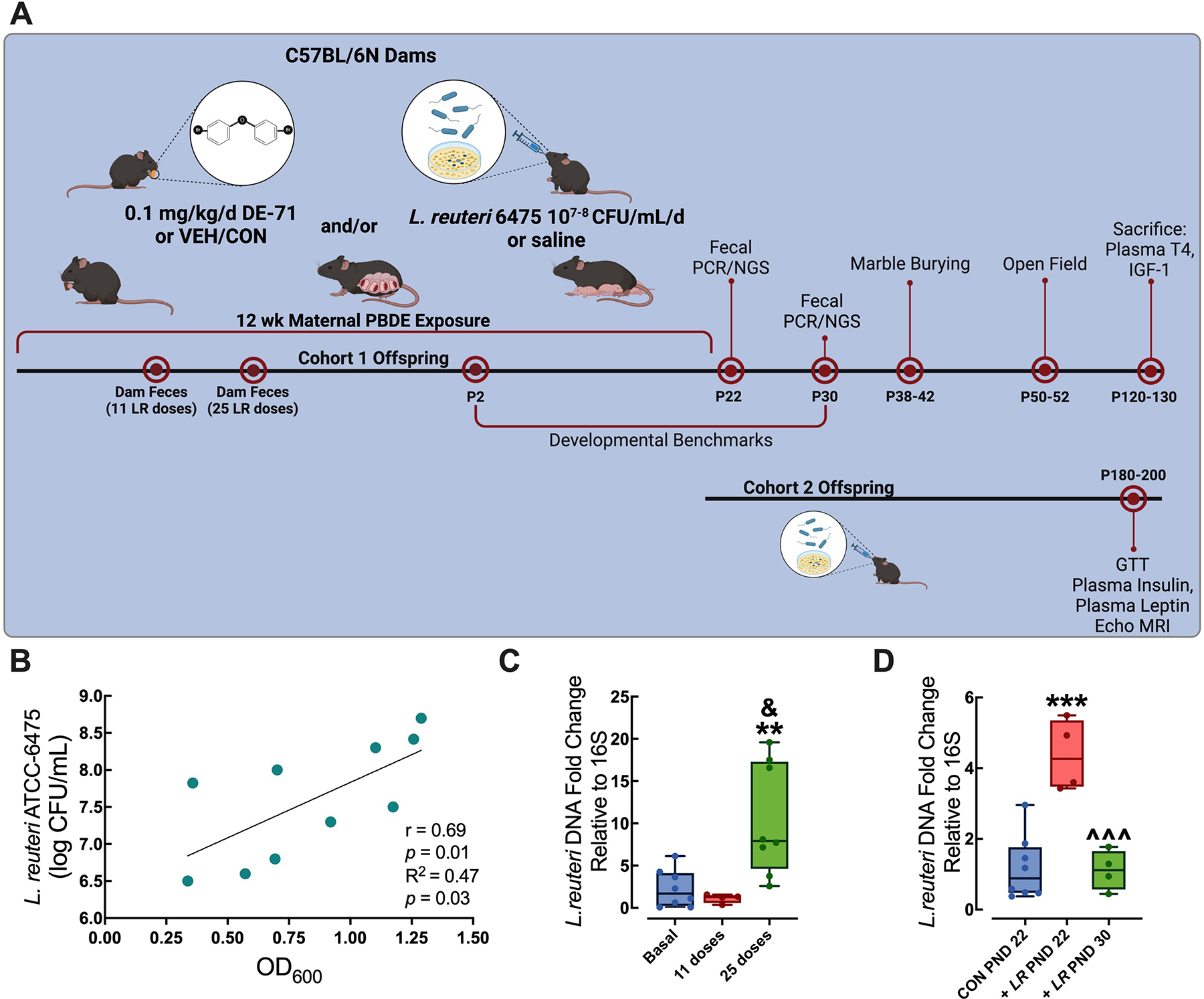
PBDE exposure paradigm and probiotic administration yifield altered gut colonization in dams and their male and female offspring. **A** Direct oral exposure of dams to a mixture of PBDE congeners, DE-71 (0.1 mg/kg/day), or vehicle control (VEH/CON), began ~ 3 weeks pre-conception and continued daily through gestation and lactation until pup weaning at P21. *L. reuteri* ATCC-6475 (~ 1 × 10^7^–10^8^ CFU/mL/day) or saline was provided to dams daily via oral gavage on the first night of mating until P21 (Cohort 1) or was additionally given directly to offspring from P22 until sacrifice (Cohort 2). Offspring developmental benchmarks wereflexamined during postnatal development starting at P2 and behavior was examined during adulthood as indicated. **B** Values for CFU/mL were acquired from cultures grown ON from bacterial suspensions at three dilutions yifielding OD_600_ values. Regression plot of the OD_600_ and log CFU/mL of *L. reuteri* ATCC-6475 cultures yifielded significant correlation and regression indicating a significant positive predictive relationship. The amount of inoculate administered was derived daily to yifield 10^7^–10^8^ CFU/mouse/day. **C**, **D** Fecal colonization of *L. reuteri* ATCC-6475 relative to general bacteria (16S) DNA was confirmed by RTqPCR in dams before (basal) and after inoculation with 11 and 25 doses (**C**) and in offspring of inoculated dams at P22 and 30 (**D**). Controls (CON) received saline. Whisker plots represent median and interquartile range representing minimum and maximum values. *statistical difference vs basal (**C**) or CON (**D**), **p < 0.01, ***p < 0.001. ŝtatistical difference vs LR offspring at P22, ^^^*p* < 0.001. ^&^statistical difference vs 11 doses, *p* < 0.05. *n*, 4–8/group (**C**, **D**). P, postnatal day; GTT, glucose tolerance test

Perinatal PBDE exposure via the dam was accomplished as described previously (Kozlova et al. 2020). In brief, virgin dams were randomly assigned to one of two exposure groups: corn oil vehicle control (VEH/CON) or 0.1 mg/kg bw per day DE-71 (Fig. 1A). A 10-week dosing regimen, chosen to model human-relevant chronic, low-level exposure (Ongono et al. 2019; Drage et al. 2019; Han et al. 2020), included ~ 4 weeks of pre-conception, plus gestation (3 weeks) and lactation (3 weeks). During the last week of the 4-week pre-conception exposure period, dams were mated with an untreated C57BL/6N male. Dams were fed oral treats (Kellogg’s Corn Flakes; Battle Creek, Michigan, USA) infused with dosing solution (2 μL/g bw) daily, except on P 0 and 1. Offspring were weaned after the lactation period at P 21 and housed in same-sex groups. Female and male offspring were studied for developmental benchmark assessment, behavioral testing and Cohort 2, which continued to receive LR into adulthood were subjected to metabolic testing and ex vivo analysis of plasma endocrine parameters. The experimental timeline is shown in Fig. 1A.

### Monospecies probiotic culture and treatment

*Limosilactobacillus reuteri* (LR, formerly *Lactobacillus reuteri* (Zheng et al. 2020)) MM4–1A (ATCC-PTA-6475; gift of BioGaia, Stockholm, Sweden) was cultured anaerobically (5% H_2_, 5% CO_2_, 90% N_2_) with deoxygenated De Man, Rogosa, and Sharpe (MRS) media (Research Products International, Mount Prospect, IL, USA) at 37 °C for 24 h. Strain identity was confirmed by quantitative polymerase chain reaction (qPCR) using DNA isolated from culture stock. To assess stock viability and quantity of colony-forming units (CFU), three concentrations of the daily inoculate were spot-plated on MRS agar plates at serial dilutions of 10^1^–10^7^-fold to enable CFU counts and correspofinding OD_600_ values measured to generate a standard curve. In Fig. 1B the regression plot of the OD_600_ and log CFU/mL of *L. reuteri* 6475 cultures yifielded a linear correlation (y = 1.497x + 6.336) with a Pearson correlation coefficient of *r* = 0.69 (*p* < 0.01) and a significant positive predictive relationship (R^2^ = 0.47, *p* = 0.03). This was used to administer ~ 1 × 10^7^–10^8^ CFU/mouse/day. In brief, daily cultures were grown to mid-log phase (OD_600_ (mean ± s.e.m) = 0.741 ± 0.12 (n = 109)). The daily inoculation volume was normalized to the equivalent of 300 μL of optical density at 600 nm (OD_600_) = 0.4 of culture, as reported (Alavi et al. 2020). Dams were orally gavaged with LR or saline daily beginning on the first night of mating until weaning of pups at P 21. Cohort 1 mice offspring received LR via mother only until weaning at P 21, while Cohort 2 offspring continued to receive LR or saline via oral gavage daily until sacrifice.

### Fecal DNA isolation and bacterial quantification qPCR

Fecal pellets were obtained from dams before (basal), and after 11 and 25 daily LR doses, and from offspring the day following weaning from mothers (P22) and at P30. DNA was isolated from mechanically disrupted (BeadBeader, Biospec, Bartlesville, OK, USA) pellets using a commercial kit (ZymoBiomics DNA Miniprep, Cat no. D4300, Zymo Research, Tustin, CA, USA). Purity and quantity of DNA was assessed by determining the optical density (OD) photometrically using 260/280 nm and 260/230 nm ratios (NanoDrop, ND-2000, ThermoFisher Scientific, Waltham, MA, USA). Oligonucleotide PCR primers for *L. reuteri* and the universally conserved bacterial 16S ribosomal RNA gene were obtained (Integrated DNA Technologies, Inc., Coralville, IA, USA) using previously published sequences (Sun et al. 2016). Primers were 97.5–100.7% efficient when detecting DNA extracted from LR cultures (Supplementary Data, Fig. 1). The following sequences were used: *L. reuter*i ATCC-6475: Forward, 5′GAAGATCAGTCGCAYTGGCCCAA-3′; Reverse, 5′-TCCATTGTGGCCGATCAG-3′. 16S bacterial primers: Forward, 5′-ACTCCTACGGGAGGCAGCAG-3′; Reverse, 5′-ATTACCGCGGCTGCTGG-3′ (Sun et al. 2016). Each DNA sample (10 ng) was ampli ed with master mix (Luna Universal M3003, New England Biolabs, Ipswich, MA, USA) using a CFX Connect thermocycler (Bio-Rad, Hercules, CA, USA). Amplification reactions were performed in 40 cycles of the following cycling protocol: initial denaturation 95 °C/1 min; per cycle 95 °C/15 s denaturation, 60 °C/30 s extension; 60–95 °C in 0.5 °C, 5 s increments melt curve analysis. Fold expression for LR was measured relative to the universal bacteria reference gene 16S, and differential gene expression was determined by comparison to dam baseline levels or saline-supplemented controls using the Pfaffl method (Pfaffl 2001).

### 16s library preparation-Next generation sequencing

Amplicon sequencing of the V4 variable region of the 16S ribosomal gene was performed as previously described (Alavi et al. 2020). In brief, 25 μL reactions containing 13.0 μL PCR-grade water (Fisher BioReagents Catalog No. BP2484–50, Pittsburgh, PA, USA), 10.0 μL Platinum Hot Start PCR Master Mix (ThermoFisher Catalog No. 13000012, Vilnius, Lithuania), 1.0 μL template DNA, and 0.5 μL forward and reverse primers (10 uM) were amplified with the following cycling protocol: 94 °C for 3 min; 30 cycles of 94 °C for 45 s, 50 °C for 60 s, and 72 °C for 90 s; and 72 °C for 10 min. An equal amount of each amplicon (~ 240 ng) was pooled for subsequent gel-isolation, silica-membrane-based purification (QIAquick PCR Purification Kit, Qiagen, Catalog No. 28704, Hilden, North Rhine-Westphalia, Germany), according to the manufacturer’s instructions. DNA sequencing (single-end 2 × 150 base) was performed using the MiSeq Reagent Nano Kit v2 (300-cycles) on the Illumina MiSeq platform (Illumina Inc., San Diego, CA, USA). Paired-end 150 nt reads were assembled, demultiplexed, and analyzed using QIIME 2 2023.9 software packages (Caporaso et al. 2010). Taxonomy was classified based on Greengenes (gg) 13 8 99% operational taxonomic units (OTUs). Each sample was rarefied to 1000 as the sampling depth for alpha and beta diversity analysis.

### Offspring early developmental milestones

Pups were transferred to a cage filled with clean bedding placed over a heating pad. Somatic growth parameters were measured on alternate days: body weight (P2–30), body and tail lengths (P2–14), eyelid opening and incisor eruption (P6–14). The following scores were used for eye opening: 0 = eyes closed, 1 = one eye open, 2 = both eyes open and for incisor eruption: 0 = no apparent growth, 1 = lower jaw eruption, 2 = both lower and upper jaw eruption. The righting reflex, a measure of latency to turn from a supine to prone position, was conducted on postnatal day (P)2–14 (Fox 1965). Observer 1 placed the pup on its back and gently held the position for 5 s. Using a stopwatch, Observer 2 evaluated pup righting, measured as the latency before all 4 limbs touched the surface in a prone position. A maximum of one min was given for each trial for a total of 3 trials per pup. Pups were allowed to rest for one min between trials.

### Marble burying

The Marble Burying (MB) test is utilized for analysis of elicited repetitive behaviors in rodents that are considered analogous to that observed in autistic individuals (Silverman et al. 2010). During MB, performed on P38–42, the test mouse was placed in the center of a polycarbonate cage (46 × 24 × 16.5 cm) containing 5 cm of bedding and allowed to interact with a 5 × 4 array of equidistant marbles for 30 min (Angoa-Pérez et al. 2013). After the test, the marbles buried in each cage were scored by 2–3 observers who were blind to treatment and a mean score was calculated. A minimum of ½ of the marble submerged in bedding was defined as being buried. Bland–Altman analysis demonstrated reliability in marble burying scores, i.e., agreement between two judges (Supplementary Data, Fig. 2).

### Open field test

Offspring (P50–52) were evaluated for locomotor activity and response to a novel environment as previously described (Burokas et al. 2017). Animals were habituated to the room 30 min prior to the test. Mice were tested during the light phase (1000–1600) at 170 LUX. Animals were placed in an open arena (40 × 32 × 24 cm) and exploration time was digitally recorded using a ceiling camera for 10 min. Mouse behavior was scored using automated video-tracking software (Ethovision XT 13, Noldus, Wageningen, the Netherlands). Time spent in the center and peripheral zones and total distance traveled in the entire arena was scored. Fecal pellets after 10 min in the arena were counted manually. Ethanol (70%) was used to remove debris and odors from apparatus between individual mouse trials.

### Plasma thyroid enzyme-linked immunosorbent assays (ELISA)

Plasma was collected from adult offspring at sacrifice (ad libitum fed state) and T4 was quantified using a commercial colorimetric enzymatic kit following manufacturer instructions (K050-H1, Arbor Assays, Ann Arbor, MI, USA). Blood was collected via cardiac puncture under isoflurane anesthesia followed by cervical dislocation, centrifuged at 2000 × g at 4 °C for 20 min and the supernatant was collected for ELISA. Samples were prepared with supplied dissociation reagent and diluted with assay buffer. The colorimetric reaction product was read as optical density at 450 nm on a plate reader (Molecular Devices, San Jose, CA, USA). Plasma T4 was quantified by interpolating optical density values at 450 nm using a 4-parameter-logarithmic standard curve of known concentrations (MyAssays, East Sussex, United Kingdom). Sensitivity and dynamic range of the assay were 0.3 ng/mL and 0.63–20 ng/mL, respectively. Of note, the thyroxine antibody in the kit had an 89% reactivity with reverse T3.

### Insulin-like growth factor ELISA

Plasma was collected from post-lactating dams and adult offspring at sacrifice (ad libitum fed state) and assayed for Insulin-like Growth Factor (IGF-1), a peptide involved in CNS growth and development that improves insulin sensitivity (Friedrich et al. 2012), using a commercial kit according to the manufacturer’s instructions (Cat.#EMIGF1, ThermoFisher Scientific, Waltham, MA, USA). Sensitivity and dynamic range of the kit were 4 pg/mL and 2.74–2,000 pg/mL, respectively. The assay has no cross-reactivity with 61 cytokines and peptides tested including leptin. The colorimetric reaction product was read as optical density at 450 nm on a plate reader (Molecular Devices). Plasma IGF-1 concentrations were determined by interpolating optical density values using a linear standard curve of known concentrations (MyAssays).

### Leptin ELISA

Plasma leptin was measured from blood collected from adult offspring at sacrifice (ad libitum fed state) using a commercial ELISA kit (Raybiotech, Cat. #ELM-Leptin-1, Norcross, GA, USA). The kit had an analytical sensitivity of 4 pg/mL in a standard range of 4–10,000 pg/mL. The colorimetric reaction product was read as optical density at 450 nm on a microplate reader. Plasma leptin concentrations were determined by interpolating absorbance values using a linear standard curve of known concentrations (MyAssays).

### Glucose tolerance tests and fasting glycemia

In order to assess glucose tolerance an glucose tolerance (GTT) was performed on Cohort 2. Mice were fasted for 11 h ON predominantly during the dark phase before they were administered a dose (2.0 g/kg b.w) of 20% dextrose in sterile saline by ip injection. Tail blood was collected at t=0 (fasting blood glucose) and at 15, 30, 60, and 120 min post-injection. Blood glucose concentrations were measured with a glucometer (One Touch Ultra 2, Lifescan Inc., Milpitas, CA, USA) and test strips, shown to be accurate to assess blood glucose concentrations in C57BL/6 J mice (Morley et al. 2018). The area under the GTT glucose curve (AUC_GTTglucose_) was calculated in Prism (GraphPad, San Diego, CA, USA). Fasting glycemia values were obtained from *t* = 0 during GTT and from another fasting session (ON for 9 h).

### Insulin ELISA

Offspring tail blood was collected at *t* = 0, 15 and 30 min post-glucose challenge during the GTT experiments and plasma insulin was assayed using the Mercodia Ultrasensitive Insulin ELISA (Cat.# 10–1249–01, Uppsala, Sweden), which had a detection limit of ≤ 0.025 ug/L in a standard range of 0.025–1.5 ug/L. Plasma total insulin was quantified by interpolating absorbance values at 450 nm using a 4-parameter-logarithmic standard curve of known concentrations (MyAssays). Animals showing no insulin response to glucose challenge were removed as outliers. The insulin:glucose ratios were obtained by dividing an individual subject’s percent change from baseline in plasma insulin over percent change from baseline in plasma glucose at *t* = 0, 15 and 30 post-glucose challenge.

### Body composition

Whole body composition (fat and lean mass) was determined in unanesthetized P 170–180 adult offspring using a quantitative magnetic resonance (QMR) system which relies on nuclear magnetic resonance technology (EchoMRI; Echo Medical Systems, Houston, TX, USA). QMR scans were performed by placing mice into a ventilated plastic cylinder with a cylindrical plastic insert added to limit movement. Body weight was measured on the day of each experiment. Duplicate QMR scans were performed with accumulation times of 2 min to determine mean fat and lean mass, expressed at percent of body weight.

### Statistical analyses

Power analyses were performed to establish sample size (GPower 3.1 (Faul et al. 2007), Universitat Dufferldorf). All statistical analyses were conducted using GraphPad Prism (GraphPad Software v9.5.1). Bars and error bars in graphs represent mean ± s.e.m unless indicated otherwise. Statistical analysis of main effects was accomplished using one-way, two-way or mixed model analysis of variance (ANOVA) with or without a repeated measures (RM) design or mixed effects model. ANOVA was followed by post hoc testing for multiple within- and between-group comparisons, using Tukey’s post hoc test when both sample sizes and variance were equal or Welch’s post hoc when variances were unequal. For two-way ANOVA, Tukey’s, Holm Sidak’s and Fisher’s least significant difference post hoc tests were used. QIIME 2 2023.9 (Caparoso et al. 2010) and R (v4.0.4 Boston, MA, USA) was used for analysis of 16S rRNA sequencing data. Group comparisons of microbial community structure were performed using permutational multivariate analysis of variance (PERMANOVA), Kruskal-Wallis or Mann–Whitney tests. Differences were considered statistically significant at *p* < 0.05. Supplementary statistical information is found in Supplemental Data 1 file.

## Results

### Gut colonization of *L*. *reuteri* in dams and their offspring after maternal treatment

Daily *L. reuteri* inoculate given to dams (~ 1 × 10^7^–10^8^ CFU/day) was obtained during the log-phase and based on correlation plot (Fig. 1B). qPCR analysis of *L. reuteri* DNA relative to general 16S bacterial DNA in dam fecal pellet homogenates showed increased expression of *L. reuteri* after 25 (*p* = 0.009), but not 11 doses compared to basal levels (*p* = 0.917) (Fig. 1C). LR treatment of dams ceased with pup weaning (P21) in Cohort 1. Fecal homogenates from Cohort 1 male and female offspring of LR-supplemented dams showed significant augmentation of LR DNA relative to saline controls (CON), an increase of 272% at P22 (*p* = 0.001). Nine days (at P30) after discontinuation of LR treatment offspring samples displayed LR levels that were significantly lower than at P22 (*p* < 0.0004) and no different than CON (*p* = 0.993) (Fig. 1D).

### PBDEs and *L*. *reuteri* change gut microbial communities

**Fig. 2:**
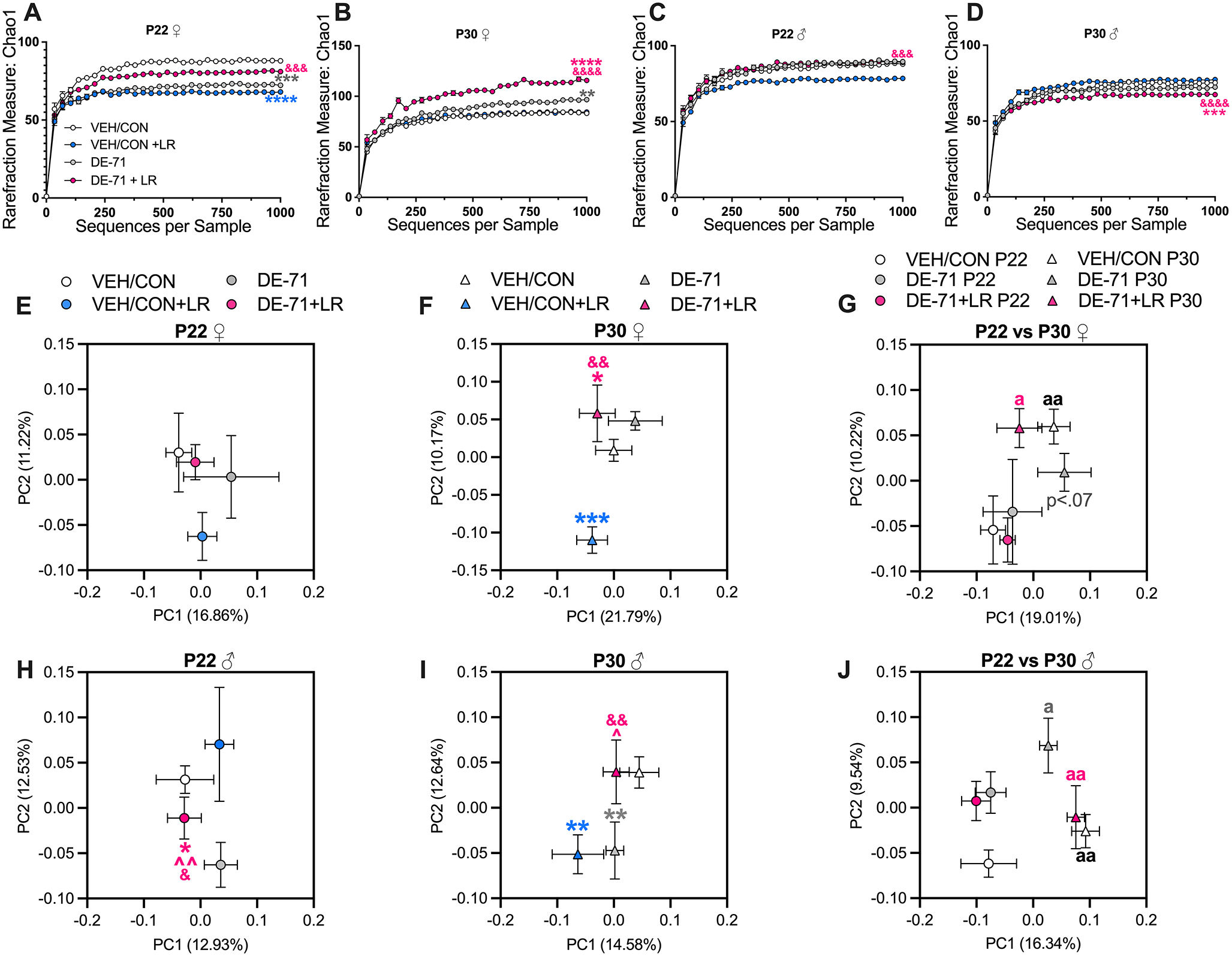
Alpha and beta diversity of microbial communities in DE-71 and LR-treated offspring. **A** P22 female Chao1 Index. **B** P30 female Chao1 Index. **C** P22 male Chao1 Index. **D** P30 male Chao1 Index. **E**–**J** Principal coordinate analysis (PCoA) plots showing across-treatment variation of the fecal microbiome in female **E**–**G** and male offspring **H**–**J** based on unweighted UniFrac distance. Percentages in parentheses indicate the proportion of variation explained by the PCoA axis. *statistical difference vs VEH/CON, *p < 0.05, **p < 0.01, ***p < 0.001 ****p < 0.0001; ŝtatistical difference vs DE-71, ^^*p* < 0.01; ^&^statistical difference vs VEH/CON + LR, ^&^*p* < 0.05, ^&&^*p* < 0.01, ^&&&^*p* < 0.001, ^&&&&^*p* < 0.0001; ^a^statistical difference vs correspofinding P22, ^a^*p* < 0.05, ^aa^*p* < 0.01. Symbol colors indicate the group being compared. *n*, 5–13/group

Given that individuals with autism spectrum disorder (ASD) display comorbidities that include microbiome dysbiosis and given that PBDEs have effects on gut microbial structure, we hypothesized that these changes accompany our previously reported DE-71-induced ASD phenotype (Kozlova et al. 2022c). We performed sequencing of the V4 region of the 16S ribosomal RNA gene in fecal samples. To estimate the richness and diversity of OTUs among samples, α-diversity values (Chao1) were calculated. Rarefaction analysis (QIIME 2) was performed from 0 to 1000 reads and results confirmed that sampling intensity was at sufficient depth, reflecting the adequacy of the sampling efforts. The Chao1 index demonstrated that DE-71 altered the richness vs VEH/CON in females at P22 (decreased, *p* < 0.001) and P30 (increased, *p* < 0.01) (Fig. 2A, B). DE-71 + LR was not different from VEH/CON in P22 females, suggesting protection of microbial richness by LR, an effect not observed at P30. In contrast, when given to VEH/CON, LR treatment decreased α-diversity (*p* < 0.0001). In males, DE-71 did not alter α-diversity (Fig. 2C, D). Combined DE-71 + LR treatment reduced species richness vs VEH/CON in P30 males (*p* < 0.001) (Fig. 2D).

Next, we compared β-diversity in gut microbial community structure using unweighted UniFrac distance matrix which considers phylogenetic distance between OTUs. Principal coordinate analysis (PCoA) and PERMANOVA revealed that all P22 female groups clustered similarly along PC1 and PC2 (Fig. 2E). In P30 females DE-71 + LR (*p* < 0.05) and VEH/CON + LR (*p* < 0.001) showed a significant difference along PC2 (10.17%) in overall community structure compared to VEH/CON (Fig. 2F). β-diversity in P22 males clustered differently in DE-71 + LR as compared to VEH/CON (*p* < 0.05) along PC2 (12.53%) (Fig. 2H). The only group that showed a substantial effect of DE-71 on β-diversity was the P30 male group which clustered differently vs VEH/CON (*p* < 0.01) along PC2 (12.64%) (Fig. 2I). In addition, β-diversity of DE-71 + LR normalized the effect of DE-71 alone (*p* < 0.05). In combination, results suggest that LR treatment produced time- and sex-dependent effects; it protected against DE-71 effects on α-diversity in P22 females and against β-diversity in P30 males.

We were interested in determining the effect of weaning the offspring from exposed mothers on offspring microbiome community structure. Therefore, we compared β-diversity in each group at weaning (P22) and 1 week after weaning (P30). In female offspring there was a significant effect of age along PC2 (10.22%) for all groups except for DE-71 (VEH/CON, *p* < 0.01 and DE-71 + LR, *p* < 0.05) (Fig. 2G), indicating that DE-71 exposure delayed maturation of microbial pro le. In contrast, significant differences for all groups were found for P30 vs P22 males along PC1 (16.34%) (VEH/CON, *p* < 0.01; DE-71, *p* < 0.05; DE-71 + LR, *p* < 0.01) (Fig. 2J). See Supplementary Data 2 for *p*- and *q*-values generated.

**Fig. 3:**
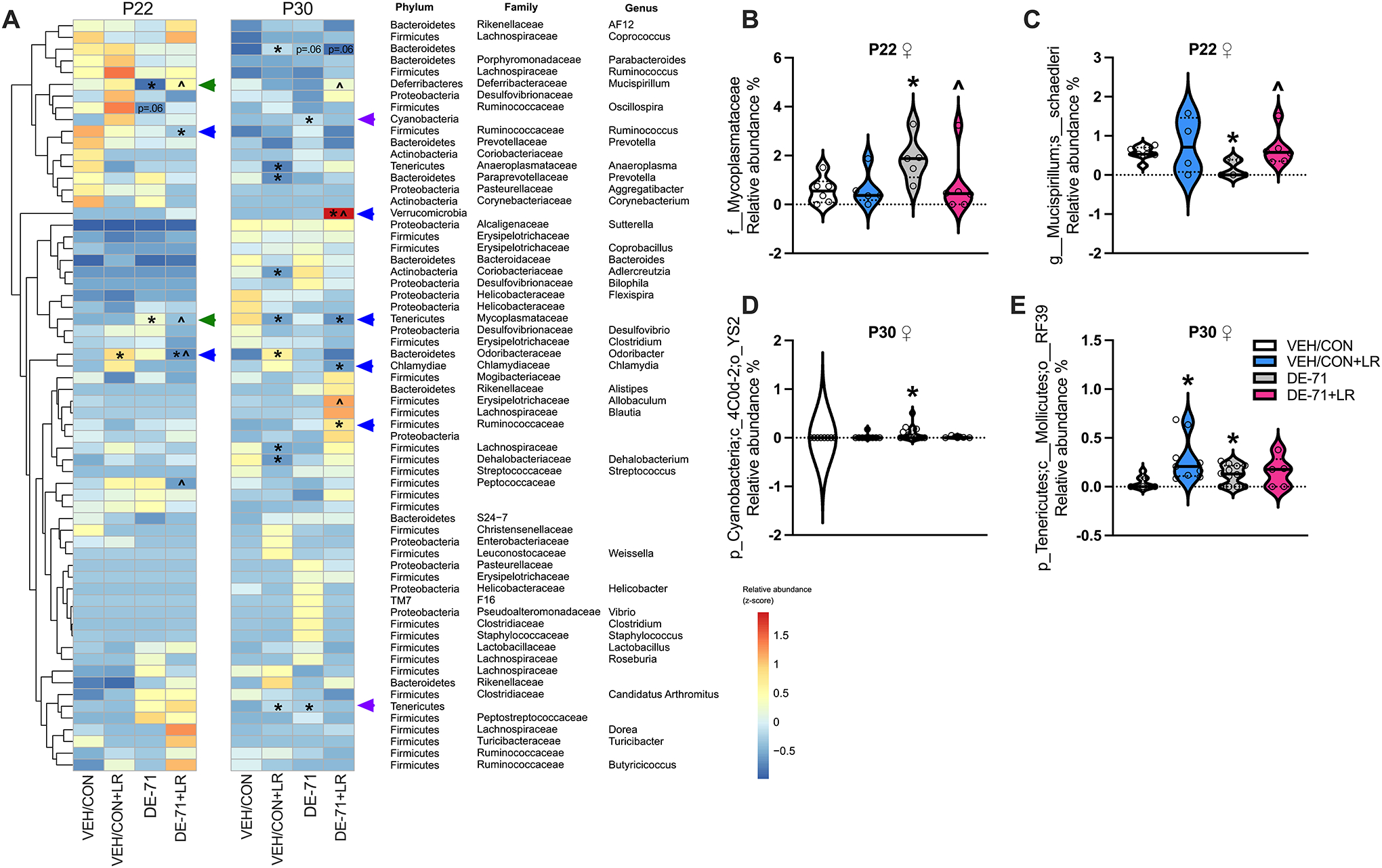
Dysbiosis produced in female offspring of dams exposed to DE-71 with or without LR supplementation. **A** Heatmap of most abundant microbial taxa identified by mapping 16S rRNA in P22 and P30 female offspring. **B**–**E** Relative abundance of taxa altered by DE-71 and/or LR at P22 (**B**,**C)** and P30 (**D**, **E)**. *statistical difference vs VEH/CON, *p < 0.05–0.001; ŝtatistical difference vs DE71, *p* < 0.05–0.001. Green arrows indicate taxa that were altered by DE-71 vs VEN/CON and normalized by additional LR supplementation. Purple arrows indicate taxa altered by DE-71, but not by DE-71 + LR. Blue arrowheads indicate taxa altered by only DE-71 + LR vs VEN/CON. *n*, 5–13/group In P22 females the heatmap shows that combined exposure to DE-71 + LR reduced the relative abundance of taxa (Fig. 3A, Blue arrows): Phylum Firmicutes Genus Ruminococcus (*p* < 0.05) and Phylum Bacteroidota Genus Odoribacter (*p* < 0.05) when compared to VEH/CON. In P30 DE-71 + LR females, taxa with reduced levels included Phylum Tenericutes Family Mycoplasmataceae (*p* < 0.05) and Phylum Chlamydiae Genus Chlamydia (*p* < 0.05).

Hierarchical clustering analysis conducted at the genus level identified 56–64 bacterial taxa in male and female offspring (Supplementary Data 3). A heatmap of z-score-transformed relative abundance revealed statistically significant group differences vs VEH/CON in female offspring (Fig. 3A; Supplementary Data 4). At P22 DE-71 exposure was associated with changes in abundance of taxa such as Phylum Tenericutes Family Mycoplasmataceae (elevated) (Fig. 3B) and Phylum Deferribacteroidota Family Deferribacteraceae Genus Mucispirillum *sp schaedleri* (reduced, *p* < 0.05) (Fig. 3C), effects that were prevented in DE-71 + LR (*p* < 0.05). These rescued taxa are indicated with green arrows on the heatmap. For P30 females DE-71, but not DE-71 + LR, increased the abundance of Phylum Cyanobacteria Order YS2 (Fig. 3D, *p* < 0.05) and Phylum Tenericutes Order RF39 (Fig. 3E, *p* < 0.05) vs VEH/CON. These taxa are indicated with purple arrows on the heatmap. Taxa that displayed increased abundance in P30 females included Phylum Firmicutes Family Ruminococcaceae (*p* < 0.05) and Phylum Verrucomicrobia (*p* < 0.05).

**Fig. 4:**
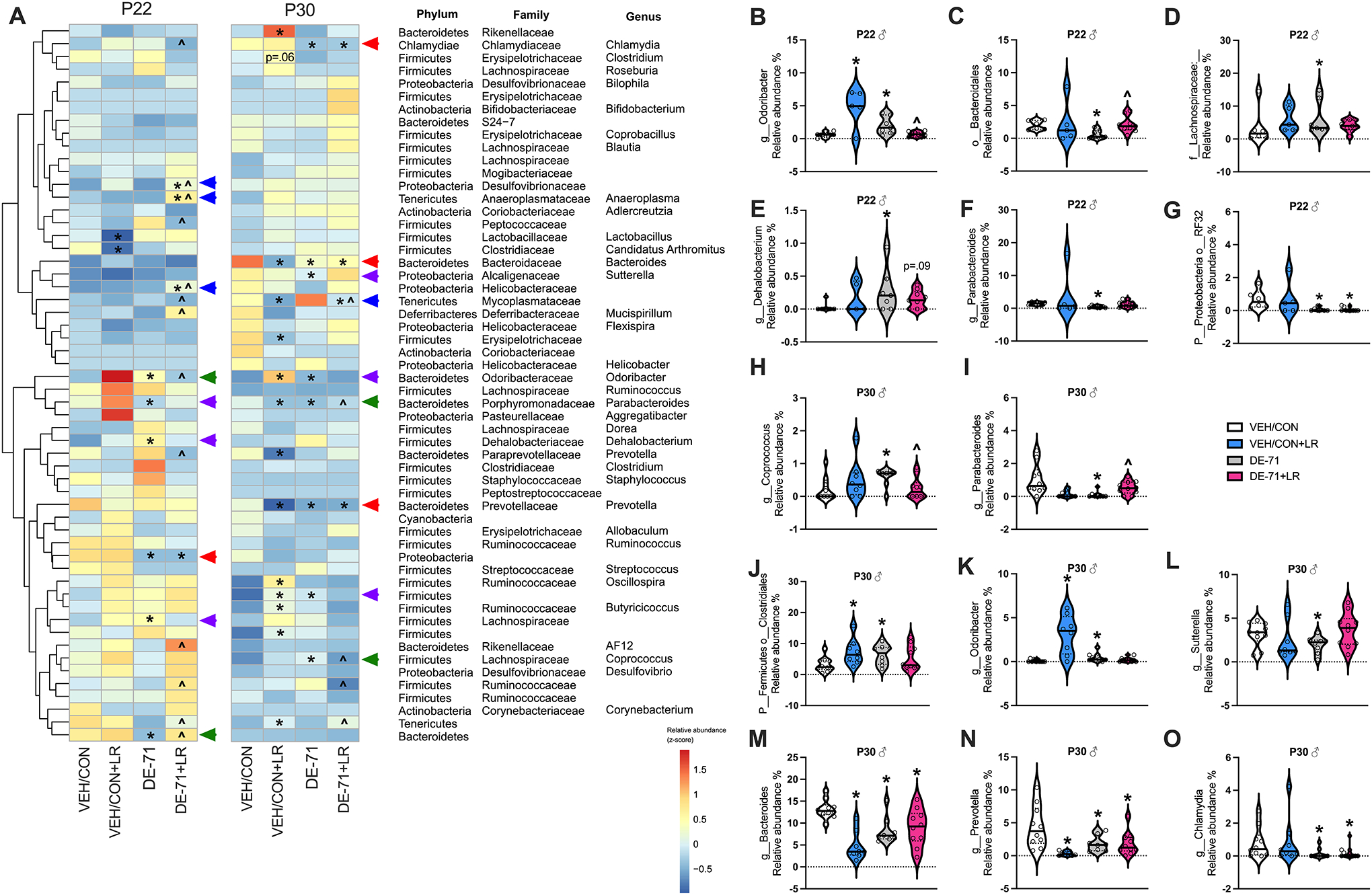
Dysbiosis produced in male offspring of dams exposed to DE-71 with or without LR supplementation. **A** Heatmap of most abundant microbial taxa identified by mapping 16S rRNA in P22 and P30 male offspring. **B**–**O** Relative abundance of taxa showing group differences at P22 (**B**–**G)** and P30 (**H**–**O)**. *statistical difference vs VEH/CON (*p < 0.05–0.001), ŝtatistical difference vs DE-71, ^*p* < 0.05–0.001. Green arrows indicate taxa that were altered by DE-71 vs VEN/CON and normalized by additional LR supplementation. Purple arrows indicate taxa altered by DE-71, but not by DE-71 + LR. Blue arrowheads indicate taxa altered by only DE-71 + LR vs VEN/CON. Red arrows indicate taxa displaying levels altered by DE-71 and DE-71 + LR vs VEH/CON. *n*, 5–13/group

Analogous data for males are displayed in the heatmap (Fig. 4A) and raw values and group comparisons can be found in Supplementary Data 3 and 4. In P22 males, compared to VEH/CON, DE-71 elevated the levels of Phylum Bacteroidota Genus Odoribactor (Fig. 4B, *p* < **0.05**) and reduced those of Phylum Bacteroidota Order Bacteroidales (Fig. 4C, *p* < **0.05**), effects that were prevented in DE-71 + LR (*p* < 0.05). These rescued taxa are indicated with green arrows on the heatmap. DE-71, but not DE-71 + LR, elevated the levels of Phylum Firmicutes Family Lachnospiraceae (Fig. 4D, *p* < **0.05**) and Phylum Firmicutes Genus Dehalobacterium when compared to VEH/CON (Fig. 4E, *p* < **0.05**). DE-71 alone also reduced the levels of Phylum Porphyromonadaceae Genus Parabacteroides (Fig. 4F, *p* < 0.01). These taxa are indicated with purple arrows on the heatmap. Phylum Proteobacterium Order RF32 was reduced in both DE-71 and DE-71 + LR (Fig. 4G, *p* = 0.007).

In P30 males, when compared to VEH/CON, DE-71 was associated with elevated levels of Phylum Firmicutes Genus Coprococcus (Fig. 4H, *p* < **0.05**), and with reduced levels of Phylum Bacteroidota Genus Parabacteroides (Fig. 4I, *p* < **0.01**), effects that were prevented in DE-71 + LR (*p* < **0.05**). These taxa are indicated with green arrows on the heatmap. DE-71, but not DE-71 + LR, increased abundance of Phylum Firmicutes Order Clostridales (Fig. 4J, *p* < **0.05**) and Phylum Bacteroidota Genus Odoribacter (Fig. 4K, p < 0.05) and decreased abundance of Phylum Proteobacteria Genus Sutterella (Fig. 4L, *p* < 0.05). These taxa are indicated with purple arrows on the heatmap. A set of taxa showed reduced abundance in both DE-71 and DE-71 + LR groups vs VEH/CON: Phylum Bacteroidota Genus Bacteroides (Fig. 4M, *p* = 0.004), Phylum Bacteroidota Genus Prevotella (Fig. 4N, *p* < 0.05) and Phylum Chlamydiae Genus Chlamydia (Fig. 4O, *p* < 0.05). These taxa are indicated with red arrows on the heatmap.

Blue arrows on the heatmap show taxa that were modified only by combined exposure to DE-71 + LR in males as compared to VEH/CON (Fig. 4A). At P22 taxa with increased abundance included Phylum Tenericutes Genus Anaeroplasma (*p* < 0.05), Phylum Proteobacteria Family Desulfovibrionaceae (*p* < 0.05), and Phylum Proteobacteria Family Helicobacteraceae (*p* < 0.05). At P30, the abundance of one taxa was decreased by combined exposure: Phylum Tenericutes Family Mycoplasmataceae (*p* < 0.01).

On average, the predominant taxa belonged to Phylum Firmicutes (Orders Clostridales and Lactobacillales) except for L-DE-71 females at P30, which had relatively greater abundance of Phylum Bacteroidota and in P30 male groups which had a more balanced ratio of Firmicutes and Bacteroidota compared to VEH/CON (Supplementary Data 3). Within the common Bacteroidota taxa, families S24–7, Prevotellaceae and Paraprevotellaceae were abundant in all groups except in female P30 L-DE-71 + LR. Abundant taxa in this group uniquely included some in Phylum Actinobacteria (Order Coriobacteriales). Other unique top-ranking orders in Phylum Tenericutes included Porphyromonas in P22 L-DE-71 + LR males and P30 L-DE-71 males, respectively. Of note, there was a lack of group difference for relative abundance of genus *Lactobacillus* in DE-71 and DE-71 + LR (Supplementary Data 1, Fig. 3).

### Sex-dependent DE-71-induced alterations in offspring growth metrics; effects of maternal LR treatment

**Fig. 5:**
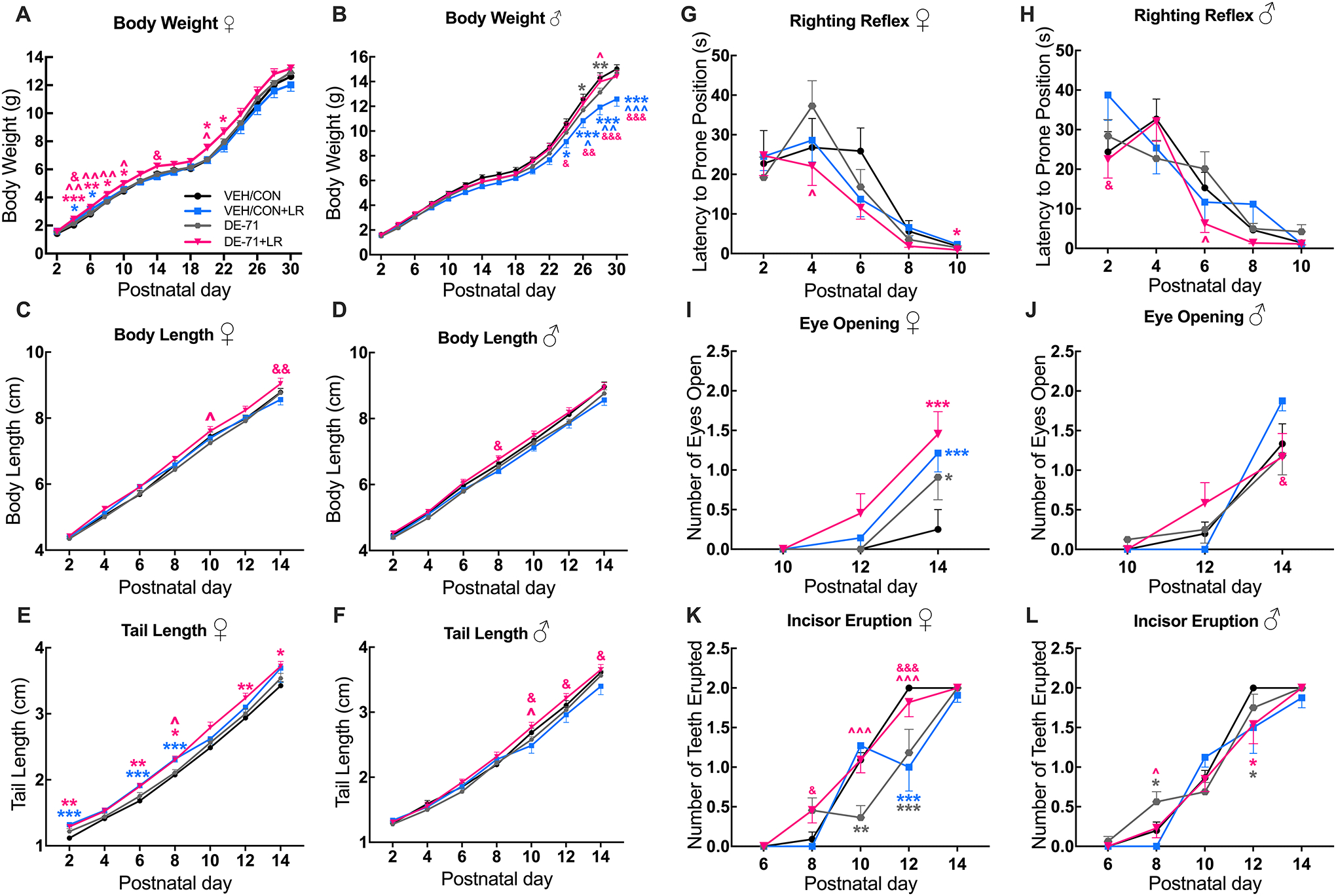
Maternal LR treatment ameliorates DE-71-induced developmental deficits in male and female offspring. Offspring received vehicle or DE-71 (0.1 mg/kg/day) with or without *L. reuteri* via their mother. Growth parameters were measured at P2-P30. **A**–**B** Body weight growth curves in females (**A**) and males (**B**). **C**–**D** Body length growth in females (**C**) and males (**D**). **E**–**F** Tail length growth in females (**E**) and males (**F**). **G**–**L** Developmental benchmarks were measured from P2 to P14. Latency to prone position in females (**G**) and males (**H**). Eye opening score in females (**I**) and males (**J**). Incisor eruption (number of teeth erupted) in females (**K**) and males (**L**). Results show that DE-71 delayed male body weight growth and female incisor eruption and LR supplementation prevented or ameliorated these deficits. Values represent mean ± s.e.m. *statistical difference vs VEH/CON, *p < 0.05, **p < 0.01, ***p < 0.001. ŝtatistical difference vs DE-71, ^*p* < 0.05, ^^*p* < 0.01, ^^^*p* < 0.001. ^&^statistical difference vs VEH/CON + LR, ^&^*p* < 0.05, ^&&^*p* < 0.01, ^&&&^*p* < 0.001. Symbol colors indicate the group being compared. Females: *n*, 8–14/group; Males: *n*, 8–19/group

Figure 5 shows offspring body weight gain during postnatal development. There was no effect of DE-71 in females. However, additional maternal LR treatment (DE-71 + LR) significantly increased female body weight on P4, 6, 8, 10 and 20 (*p* < 0.001, *p* < 0.01, *p* < 0.05, *p* < 0.05, *p* < 0.05, respectively). Maternal LR supplementation also increased female body weight in VEH/CON on P4 and 6 (*p* < 0.05) (Fig. 5A). In males, a significant decrease in body weight was observed in DE-71, but not DE-71 + LR, relative to VEH/CON on P26, 28 (*p* < 0.05, *p* < 0.01, respectively; Fig. 5B). LR supplementation had opposite effects on mean body weight in male DE-71 and VEH/CON groups; body weight in DE-71 + LR male mice was increased at P28 (*p* < 0.05) and reduced in VEH/CON + LR male mice on P24–30 (*p* < 0.05, *p* < 0.001, *p* < 0.001, *p* < 0.001, respectively).

No major effects of treatment were observed on the time-dependent increase in mean body length for female or male offspring. However, LR treatment increased body length in DE-71 + LR vs DE-71 in females at P10 (*p* < 0.05) as well as vs VEH/CON + LR at P14 (*p* < 0.01) (Fig. 5C). In males, mean body length was greater in DE-71 + LR vs VEH/CON + LR at P8 (*p* < 0.01) (Fig. 5D), indicating an interaction between LR treatment and DE-71 exposure.

There was no main effect of DE-71 exposure in males or females on tail length as compared to VEH/CON. However, in females, combined treatment of DE-71 + LR increased mean tail length vs DE-71 on P8 (*p* < 0.05). However, tail length was abnormal since mean value in DE-71 + LR was greater vs VEH/CON at P2, 6, 8, 12, 14 (*p* < 0.01, *p* < 0.01, *p* < 0.05, *p* < 0.01, *p* < 0.05, respectively). LR treatment in controls (VEH/CON + LR) also increased mean tail length vs VEH/CON on P2, 6, 8 (*p* < 0.001; Fig. 5E). In male offspring LR treatment had the greatest positive effect on mean tail length when combined with DE-71 (DE-71 + LR), yielding significantly greater mean values vs DE-71 at P10 (*p* < 0.05) and vs VEH/CON + LR on P10, 12, 14 (*p* < 0.05) (Fig. 5F).

### Effects of DE-71 exposure and maternal LR treatment on righting reflex, eye opening and incisor eruption

We also measured other developmental benchmarks in offspring. Compared to VEH/CON, there was no effect of DE-71 treatment on the righting reflex in male or female offspring. However, Fig. 5G shows that LR supplementation improved mean latency of righting reflex in females: DE-71 + LR vs VEH/CON at P10 (*p* < 0.05) and vs DE-71 at P4 (*p* < 0.05). Similarly, in males, LR supplementation lowered mean latency of righting reflex: DE-71 + LR vs DE-71 at P6 (*p* < 0.05) (Fig. 5H). Moreover, righting reflex latency was shorter for DE-71 + LR vs VEH/CON + LR at P2 (*p* < 0.05).

For eye opening, all female treatment groups displayed expedited eye opening at P14 when compared to VEH/CON, which contained the most delayed phenotype: DE-71 (*p* < 0.05), DE-71 + LR (*p* < 0.001), VEH/CON + LR (*p* < 0.001) (Fig. 5I). Figure 5J shows minimal group differences in males.

Incisor eruption in females was delayed in DE-71 (but not DE-71 + LR) at P10 and P12 as compared to VEH/CON (*p* < 0.01, *p* < 0.001, respectively) (Fig. 5K). LR treatment given to controls (VEH/CON + LR) delayed incisor eruption at P12 (*p* < 0.001). In male offspring DE-71 expedited incisor eruption vs VEH/CON at P8 (*p* < 0.05) (Fig. 5L). This was normalized in DE-71 + LR vs DE-71 (*p* < 0.05).

### Maternal LR treatment rescued exaggerated digging in DE-71-exposed female but not male offspring

**Fig. 6:**
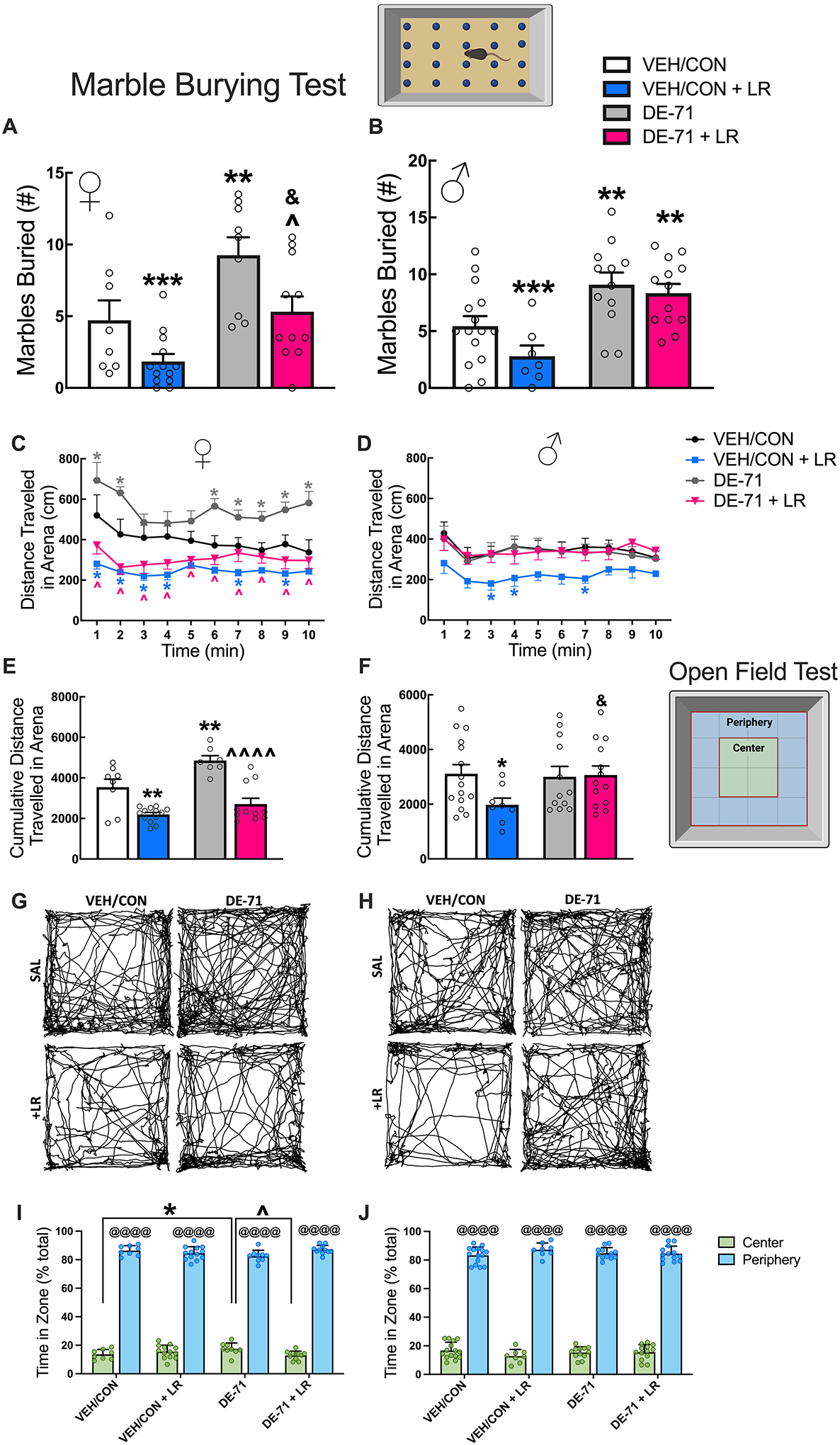
Maternal LR treatment prevents DE-71-triggered repetitive behavior in exposed female but not male offspring. Offspring received vehicle or DE-71 (0.1 mg/kg/day) with or without *L. reuteri* via their mother. **A** and **B** Adult offspring were subjected to a marble burying test and the number of marbles buried were counted after 30 min. Marble burying score for females (**A**) and males (**B**). **C**–**J** Adult offspring were subjected to an open field test for 10 min. Total distance traveled in 1 min intervals by female (**C**) and male offspring (**D**). Cumulative distance traveled in the arena for females (**E**) and males (**F**). Representative raster plots of distance traveled in females (**G**) and males (**H**). Distance traveled by zone (center vs periphery) in females (**I**) and males (**J**). Values represent mean ± s.e.m. *statistical difference vs VEH/CON (**p < 0.01, ***p < 0.001 in A,B,E,F,I; *p < 0.05–0.0001 in C,D), ŝtatistical difference vs DE-71 (^*p* < 0.05 in A, ^*p* < 0.0001 in C, ^^^^*p* < 0.0001 in E), ^&^statistical difference vs VEH/CON + LR (^&^*p* < 0.05). ^@^statistical difference vs center zone, ^@@@@^*p* < 0.0001. Symbol colors indicate the group being compared. *n*, 8–13/group (**A**) and 7–15/group (**B**); *n*, 8–15 mice/group (**C**–**J**)

Repetitive behavior such as digging was assessed using a marble burying test. DE-71 treated female offspring buried a significantly greater number of marbles relative to VEH/CON (*p* < 0.01) and DE-71 + LR (*p* < 0.05) (Fig. 6A). In contrast, male offspring in both DE-71 (*p* < 0.01) and DE-71 + LR groups (*p* < 0.01) showed exaggerated marble burying vs VEH/CON (Fig. 6B). Therefore, LR supplementation normalized the DE-71-induced repetitive behavior only in females.

### Maternal LR treatment mitigated DE-71-induced hyperactivity in female offspring

Scatter plots of distance traveled over time in an open field arena by female offspring indicated hyperactivity induced by DE-71 vs VEH/CON at *t* = 1, 2, 6–10 min (*p* < 0.05–0.0001) (Fig. 6C). This was ameliorated by additional treatment with LR (DE-71 + LR) at all time points (*p* < 0.01–0.0001), suggesting that LR treatment decreased exploratory behavior augmented by DE-71 exposure. VEH/CON + LR also traveled less distance vs VEH/CON at *t* = 1,2,3,4,7,9 min (*p* < 0.05–0.0001). In males, there was no effect of DE-71 on distance traveled and LR treatment reduced distance traveled in VEH/CON at *t* = 3,4,7 min (*p* < 0.05) (Fig. 6D). Group differences were found when examining cumulative distance traveled over 10 min (Fig. 6 E,F). In both male and female offspring, LR treatment (VEH/CON + LR) produced a significant reduction in cumulative distance traveled vs VEH/CON (females *p* < 0.01, males *p* < 0.05) (Fig. 6E, F). Only DE-71 females showed significant hyperactivity that was mitigated in DE-71 + LR relative to DE-71 (*p* < 0.0001). Representative group raster plots reflect hyperactivity shown by DE-71 females and less by both LR groups (Fig. 6 G, H).

Distance traveled in an open field by zone was examined to assess anxiety behavior; mice normally show a preference for peripheral zone (*p* < 0.0001). Using percent time in the center zone there was a significant effect of exposure and zone for female offspring, with less anxiety shown by DE-71 (*p* < 0.05) vs VEH/CON (Fig. 6I), an effect that was normalized by DE-71 + LR (*p* < 0.05). In males, there was a significant effect of zone only. For all mice, there were no group differences in the total number of fecal boli defecated in the open field chamber over 10 min, another measure of anxiety (Supplementary Data 1, Fig. 4).

### Maternal LR treatment protected against DE-71 effects on glucose tolerance in a sex-dependent manner

**Fig. 7:**
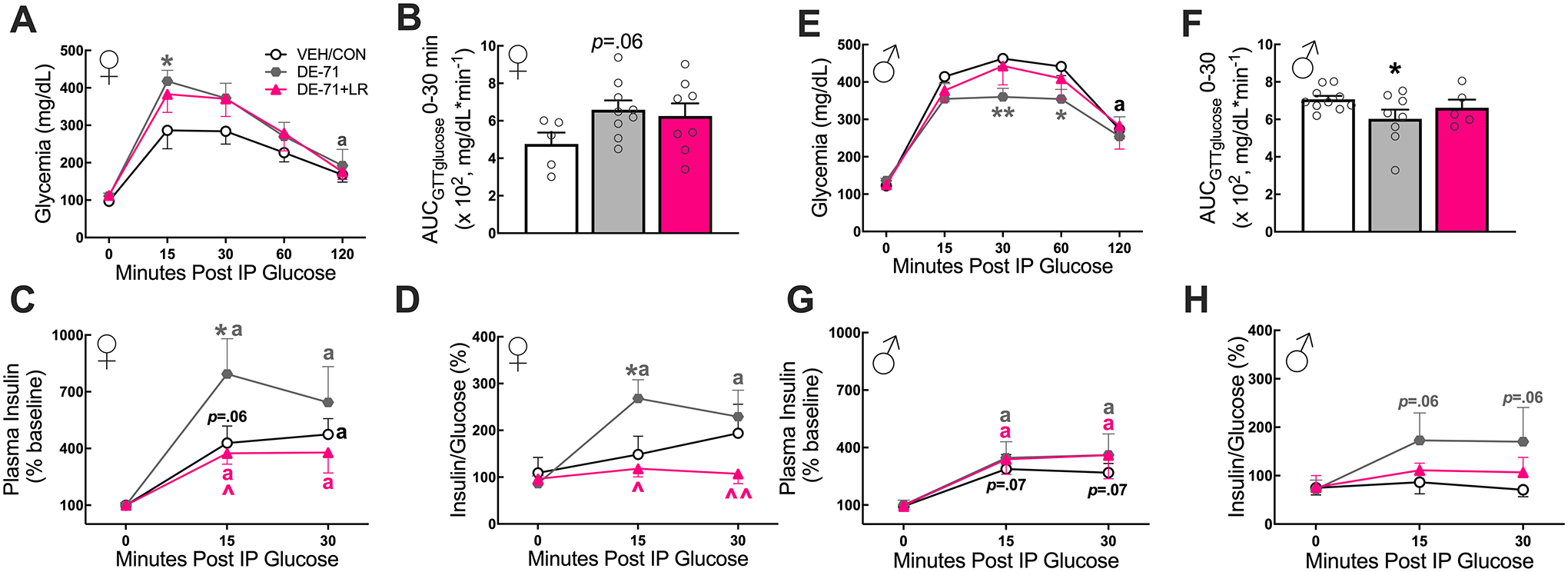
Maternal LR treatment reduced DE-71-induced glucose metabolic reprogramming in a sex-dependent manner. Offspring received vehicle or DE-71 with or without *L. reuteri* via their mother. Adult offspring were fasted for 11 h ON and tail blood was sampled for glucose before (*t* = 0 min) and after (*t* = 15, 30, 60 and 120 min) i.p. injection of 2.0 g/kg glucose during a GTT. **A**–**D** Cohort 2 Female offspring. **E**–**H** Cohort 2 male offspring. **A**, **E** Absolute blood glucose concentrations taken during GTT (GTT_glucose_). **B**, **F** Mean values for the integrated area under the GTT glucose curve (AUC_IPGTTglucose_) during *t* = 0–30 min. **C**, **G** Plasma insulin levels during GTT. **D**, **H** Insulin-to-glucose ratio during GTT. Values represent mean ± s.e.m. *statistical difference vs VEH/CON, *p < 0.05, **p < 0.01. ŝtatistical difference vs DE-71, ^*p* < 0.05, ^^*p* < 0.01. ^a^statistical difference from baseline at *t* = 120 min in panels A (*p* < 0.05) and E (*p* < 0.001) and at *t* = 15 or *t* = 30 in panels C (*p* < 0.05–0.0001), D (*p* < 0.01), and G (*p* < 0.05–0.01). Symbol colors represent the groups showing difference. *p* values placed on graphs in panels B, C, G and H indicate apparent change from correspofinding control group or within group baseline. *n*, 5–10/group

Having previously reported metabolic alterations in mice perinatally exposed to DE-71 (Kozlova et al. 2020, 2022a, 2023), we examined the effect of prolonged LR supplementation on glucoregulation in Cohort 2. Glucose challenge produced sex-dependent glycemia responses. Specifically, in females, glycemia was significantly greater at 15–60 min post-glucose injection when compared to baseline (*p* < 0.01-*p* < 0.001) and returned to normal by 120 min for all groups except DE-71 (*p* < 0.05) (Fig. 7A). Between group comparisons indicated that female DE-71, but not DE-71 + LR, showed glycemia that was greater vs VEH/CON at *t* = 15 (*p* < 0.05). Using the area under the glucose curve, AUC_GTTglucose_, to represent glycemia over the first 30 min post-glucose injection, the DE-71, but not DE-71 + LR group, showed an apparent greater peak vs VEH/CON (*p* = 0.06) (Fig. 7B). When glycemia was represented as percent of baseline, there were no group differences in magnitude at any time point (Supplementary Data 1, Fig. 5).

Insulin levels obtained during GTT showed an apparent or significant rise at *t* = 15 (p = 0.06) and *t* = 30 (p < 0.05), respectively, vs baseline in all female groups (Fig. 7C). Notably, DE-71 females also showed an elevated insulin-to-glucose ratio at *t* = 15 (*p* < 0.01) (Fig. 7D). Between group comparisons indicated greater elevation in peak insulin at *t* = 15 for DE-71 vs VEH/CON (*p* < 0.05); this was normalized in DE-71 + LR (*p* < 0.05). Similar group effects were found for mean values of insulin-to-glucose ratio at *t* = 15 (*p* < 0.05); this was normalized in DE-71 + LR vs DE-71 at t = 15 (*p* < 0.05) and t = 30 (p < 0.01). DE-71, but not DE-71 + LR males, displayed better glucose tolerance, e.g., they showed a significant decrease in glycemia post-glucose challenge, vs VEH/CON at *t* = 30 (*p* < 0.01) and *t* = 60 (*p* < 0.05) (Fig. 7E). The corresponding AUC_GTTglucose_ over 0–30 min post-glucose injection showed reduced glycemia in DE-71 males (Fig. 7F). In males, as expected, there were significant effects of treatment and time on insulin response to glucose injection. Groups showed an apparent rise in insulin (VEH/CON, *p* = 0.07) or a significant rise (DE-71, p < 0.05 and DE-71 + LR, *p* < 0.05) at *t* = 15 and *t* = 30 vs baseline values. Mean values for insulin-to-glucose ratio yielded no significant main effects of time or treatment in males (Fig. 7H). However, posthoc tests indicated an apparent increase in insulin:glucose in DE-71, but not DE-71 + LR, males (*p* = 0.06). Similar GTT results were obtained using glycemia values as a percent of baseline (Supplementary Data 1, Fig. 5).

### DE-71 and LR effects on fasting glucose, body weight, body composition and plasma leptin, T4 and IGF-1

We measured other parameters associated with Cohort 2 growth and glucose metabolism. Supplementary Data 1, Table 1 shows mean group values for fasting blood glucose, and fed-state body weight, body composition and plasma leptin. Fasting glycemia was normal in DE-71 females and males as compared to VEH/CON. After 9 h fasting, the glycemia in females was lower in DE-71 + LR vs DE-71 (*p* < 0.05). For body weight and composition (fat and lean mass), there were no group differences found in females or males. For fed-state leptin levels only DE-71 females showed reduced levels as compared to VEH/CON (*p* < 0.05). In adult offspring of Cohort 1, no group differences were observed in plasma T4 or IGF-1 (Supplementary Data 1, Fig. 6).

### Summary of parameters affected by DE-71 with or without maternal LR treatment

Table 1 shows a summary of developmental benchmarks and adult physiology affected by maternal DE-71 exposure with/or without LR supplementation.

## Discussion

There is growing concern that maternal environmental stressors during early life have long-term detrimental effects on offspring health. In this work, we report novel information about the prevention potential of maternal probiotics on postnatal and adult physiological, morphological and behavioral parameters disrupted by PBDEs. We show that chronic maternal exposure to a penta-mixture of PBDEs, DE-71, administered at environmental relevant levels, interferes with developmental benchmarks, neurobehavior and metabolic homeostasis in a sex-dependent manner. Maternal supplementation with *L. reuteri* ATCC-6475 (LR) alters the timing of incisor eruption in both sexes, normalizes body weight gain in male offspring, and normalizes adult behavior and diabetic markers in female offspring exposed to DE-71.

The maternal DE-71 transfer model used in our study is humanly relevant and of translational value, as the resulting BDE congener profiles in offspring brains are within 10X of median levels detected and similar to the maximum levels found in biomonitored human breast milk and toddler plasma (Costa and Giordano 2007; Kozlova et al. 2022c). Moreover, administration of live LR cultures to DE-71-exposed mothers provided gut colonization in early postnatal offspring. Lactational transfer through P21 was effective in colonizing suckling offspring after which fecal LR levels dropped (Cohort 1), suggesting a constant supply is needed for colonization. Nevertheless, LR may have still resided in the proximal gut but reduced shedding prevented fecal detection (Walter et al. 2007). Indeed, LR colonization requires mucosal adhesion of bacteria, especially for *Lactobacillus* species (Li et al. 2008). Notably, DE-71-exposed female offspring that received maternal LR (Cohort 1) displayed normal neurobehavior in adulthood, suggesting that the beneficial effects of LR occur during a critical developmental window when toxicant-induced neurotoxicity is most detrimental (Dingemans et al. 2007, 2011; Herbstman and Mall 2014).

One of the main findings of our study was that perinatal DE-71 delayed body weight gain during early postnatal development in male but not female offspring. However, we cannot rule out the possibility that DE-71 exposure lowered female offspring body weight at later time points than measured here, as described for P35–60 rat offspring (Kodavanti et al. 2010; Kozlova et al. 2020). Other animal studies report the effects of single BDE congeners on body weight with increases (Gee and Moser 2008; Li et al. 2021a), decreases (Chao et al. 2007; Kim et al. 2015; Ta et al. 2011), and no changes (Koenig et al. 2012; de-Miranda et al. 2016). For example, low dose (0.2 mg/kg bw) perinatal BDE-47 exposure increased body weight and length in male and female rat pups (Suvorov et al. 2009a). Human studies have found an inverse association between maternal PBDE burden and birth weight (Harley et al. 2011), body length (Chao et al. 2007), and adverse birth outcomes (Wu et al. 2010). However, some studies report contradictory results (Lopez-Espinosa et al. 2015; Mazdai et al. 2003; Miranda et al. 2015 and Stasinska et al. 2014). Importantly, we show for the first time that maternal LR supplementation normalized body weight gain in DE-71-exposed male offspring. This finding may have clinical translational consequences of medical value since low birth anthropometric measurements contribute to 60–80% of all neonatal deaths annually, with co-morbidities ranging from neurodevelopmental/motor outcomes to chronic metabolic dysfunction as adults (Liao et al. 2020; Upadhyay et al. 2019; Xiao et al. 2010). Therefore, maternal LR probiotic treatment may be leveraged early to prevent deficient growth in unborn and developing children, which may reduce the risk of NDDs associated with reduced birth weight, such as cerebral palsy, autism, schizophrenia, and attention-deficit/hyperactivity disorder (ADHD) (Cortese et al. 2021).

Developmental abnormalities produced by DE-71 included delayed incisor eruption in P10–12 females and expedited eruption in P8 males. Importantly, maternal LR supplementation normalized these changes. DE-71 also expedited eye opening in females, a well-known developmental milestone in mice that relies, in part, on the reorganization of the visual cortex (Rochefort et al. 2009; Sur et al. 2013) However, LR supplementation further advanced eye opening in P14 females. The penta-PBDE mixture, DE-71, appears more harmful to postnatal development than single congeners used by others. For example, neither BDE-99 (2,2′,4,4,5-pentabromodiphenylether) at doses of 0.6 and 6 mg/kg/d had effects on righting reflex, eye opening, incisor eruption, and body weight in exposed female CD-1 Swiss mice (Branchi et al. 2002) and BDE-47 (2,2′,4,4′-tetrabromodiphenyl ether) at 1, 10, 30 mg/kg given in a single dose at P10 did not affect eye opening and body weight in male C57BL/6 mice (Gee and Moser 2008). Studies on preterm human infants have also noted that probiotic treatment can improve short-term weight gain without effects on neurodevelopmental outcomes (Panchal et al. 2023).

Marble burying in rodents is used to assess autism-relevant restricted and repetitive behavior (Thomas et al. 2009). We previously reported exaggerated digging, in addition to social behavior deficits, in DE-71-exposed adult female mice offspring (Kozlova et al. 2022c). Here, we obtained similar findings for DE-71-exposed male and female offspring. Consistent with our findings, BDE-209 (0.12 ng/day) (Chen et al. 2019) and BDE-47 (Li et al. 2021b) increased marble burying in male mice, suggesting common effects of BDE congeners. More importantly, we found that maternal LR supplementation normalized exaggerated digging displayed by DE-71 females but not males. In other models of autism, such as maternal high-fat diet (HFD), direct administration of *L. reuteri* ATCC-6475 to weanling males (P21 for 4 wk) failed to reverse repetitive behavior (Buffington et al. 2016) but was effective in adult males and females in a monogenetic *Shank3* ASD model (Tabouy et al. 2018). Importantly, direct LR treatment to male mice weanlings can reverse other ASD domains such as social behavior in monogenetic (*Shank3, Catnap2)*, idiopathic (BTBR), and valproic acid rodent models (Sgritta et al. 2019). In a recent human trial, LR increased social functioning without altering autistic severity or repetitive behaviors (Mazzone et al. 2024), indicating specificity of action by LR depending on the autistic phenotype.

Studies in humans have raised concern that PBDEs may cause hyperactivity in children (Vuong et al. 2017), acting as environmental risk factors related to motor abnormalities in ASD, ADHD, and other NDDs (Dougnon and Matsui 2022). In the current study, DE-71-exposed adult female mice displayed increased locomotor activity (hyperactivity) during an OFT. While we previously reported no DE-71 effects on locomotor activity in younger P30 female offspring (Kozlova et al. 2022c), our current findings in adult females are supported by single congener studies using perinatal exposure to BDE-47 and BDE-99 (Branchi et al. 2002; Suvorov et al. 2009b; Qiu et al. 2022). For a review see (Costa and Giordano 2007). As hypothesized, we observed that maternal LR supplementation rescued normal locomotor activity and anxiety in females. Several studies of monogenetic (*Shank3, Cntnap2)* or environmental (maternal HFD) ASD report no benefit of LR in reducing hyperactivity or anxiety in male or female offspring (Buffington et al. 2016, 2021; Sgritta et al. 2019; Tabouy et al. 2018). Our results, demonstrating that developmental LR supplementation reduced hyperactivity and anxiety in DE-71 females, highlight the potential benefit of maternal LR therapy to a toxicant model of autism.

Metabolic measurements showed abnormal sex-dependent glucoregulatory responses produced by DE-71 exposure. In agreement with our previous published reports (Kozlova et al. 2020, 2023), DE-71-exposed female offspring displayed glucose intolerance and significantly greater insulin-to-glucose ratios, which is used as an index of insulin resistance, when compared to the control group. A major finding of this study is that maternal LR supplementation can protect against this female-specific phenotype caused by early-life exposure to DE-71. In adult male offspring, LR therapy normalized the *improved* glucose tolerance produced by DE-71. Previous studies have reported anti-diabetic properties of specific *Lactobactillus* strains (but also *Saccharomycetes*, and *Bifidobacterium bifidumi*) (Bagarolli et al. 2017; Wang et al. 2020). For example, *L. reuteri GL-104* decreased fasting blood glucose and lipid profiles, and improved glucose tolerance in genetic diabetic-obese (db/db) mice with a mutation in the leptin receptor (Hsieh et al. 2020b) Additionally, *L. reuteri GMNL-263* improved insulin responses and ameliorated hepatic steatosis in high fructose-fed rats (Hsieh et al. 2013). *L. reuteri* ATCC (strain PTA 4659) can also reduce serum insulin levels in adult mice with metabolic syndrome (Apoe^−/−^ mice fed a high-fat diet) (Fåk and Bäckhed 2012). This benefit was associated with increased hepatic expression of carnitine palmitoyltransferase 1a (*Cpt1a*), proposed to combat hyperglycemia, insulin resistance, and metabolic syndrome (Schreurs et al. 2010). LR benefits may also be imparted via its high antioxidant capacity and anti-inflammatory actions, which protect insulin-secreting cells and gut function. These effects are shared with other *Lactobacillus* strains (Chen and Zhang 2023). In DE-71 females, LR therapy also mitigated DE-71-triggered reduction of another key metabolic hormone, leptin, which may have contributed to exaggerated insulin-to-glucose response (Paz-Filho et al. 2012). These and other potential mechanisms underlying the anti-diabetic effects of LR must be investigated further. Nevertheless, our present findings are the first to demonstrate that maternal probiotic therapy with LR can prevent the dysregulation of glucose homeostasis in offspring produced by early-life PBDE-induced reprogramming. These findings support the effective use of maternal transfer of probiotics against obesity and metabolic syndrome, as in maternal HFD and Bisphenol A models studied, respectively (for review see (Huang et al. 2022; Mu et al. 2018).

In a clinical study, a novel prescription containing 8 probiotic species from the genera *Lactobacillus, Bifidobacterium, Streptococcus, and Saccharomyces* has been used to improve glucose metabolism in subjects with pre-diabetes and early type II diabetes mellitus (T2DM) (Palacios et al. 2017). Certain probiotic species such as *Lactobacillus and Bifidobacterium* can improve insulin sensitivity, inflammatory markers, and lipid profiles in obese, T2DM, and dyslipidemic subjects (Asemi et al. 2014; Bukowska et al. 1998; Ejtahed et al. 2011; Mobini et al. 2017). Ingestion of *L. reuteri* capsules modulates secretion of insulin, C-peptide, and proglucagon-derived gut peptides (Simon et al. 2015). Three months of oral consumption of the live *L. reuteri* ADR-1, but not the heat-killed *L. reuteri* ADR-3, reduces HbA1c and serum cholesterol, and is associated with at least an eightfold increase in fecal *L. reuteri* (Hsieh et al. 2020a).

PBDE body burdens have been associated with altered adult (Cruz et al. 2020; Qiu et al. 2022), maternal (Gao et al. 2021), and childhood microbiome profiles (Iszatt et al. 2019; Laue et al. 2019) in humans and animals. In our study, altered relative abundance of OTUs associated with DE-71 exposure manifested as reduction of specific taxa in Deferribacterota in females and Bacteroidota, Chlamydiae, and Proteobacteria in males. Taxa showing elevated abundance belonged to Tenericutes and Cyanobacteria in females and Firmicutes and Bacteroidota in males. Changes in the relative abundance of similar taxa, i.e., Firmicutes (elevated) and Bacteroidota (reduced), have been associated with increased anxiety and hyperactivity in adult female rats exposed to PBDE-47 (10 mg/kg) (Qiu et al. 2022). Taxa in Proteobacteria and Deferribacterota were also more abundant. They also found BDE-47-induced changes in Euryarchaeota (greater) and Saccharibacteria (reduced). Of note, we did not detect altered abundance in Genus Lactobacillus in DE-71 exposed offspring, unlike results of a previous rodent study using PBDE-47 (Scoville et al. 2019) and an epidemiological report showing a positive association with PBDE-28 (Iszatt et al. 2019). These discrepancies may be due to different actions of BDE congeners in DE-71, or the species, sex, or age of the host.

Mounting evidence has confirmed altered gut microbial composition in children suffering from ASD and manipulation of symptoms through probiotic intervention (Kang et al. 2019; Sivamaruthi et al. 2020). *Lactobacillus* probiotic therapy used in mouse models of neurodevelopmental disorders can resolve deficits associated with an autistic phenotype whether or not abnormally low levels of fecal LR were present (Buffington et al. 2016; Sgritta et al. 2019). Of particular note, we found that supplementation with LR normalized the abnormal repetitive, anxiety, and hyperactive behavior produced by perinatal DE-71. Moreover, mice displaying DE-71-triggered developmental and neurobehavioral deficits also displayed LR-reversible gut dysbiosis. Using 16S rRNA sequencing of fecal samples we discovered that LR protected against DE-71-reduced microbial richness (α-diversity) in P22 females. The significance of this is linked to the fact that lessened complexity in gut microbiome, especially in early life, is associated with adult disease (Clarke et al. 2019; Tamburini et al. 2016). Of the 2 taxa that were significantly modified by DE-71 in P22 females, both were normalized by LR supplementation: Phylum Tenericutes Family Mycoplasmataceae (prevented increase), which is associated with S100B, an indicator for brain damage and blood brain barrier disruption (Qing et al. 2021), and Phylum Deferribacteraceae Genera Mucispirillum *sp schaedleri* (prevented reduction). In contrast, in P22 males LR normalized only 2 of 6 modified taxa: DE-71-triggered reduction in relative abundance of Phylum Bacteroidota Order Bacteroidales and the rise in Phylum Bacteroidota Genus Odoribacter, a short-chain fatty acid-producing bacteria within the human intestinal microbiota that improves glucose control and reduces inflammation (Hiippala et al. 2020; Huber-Ruano et al. 2022). This was associated with the normalization of adverse effects of DE-71 on β-diversity and delayed body weight gain. In P30 males LR normalized DE-71-elevated levels of Phylum Firmicutes Genus Coprococcus, which have been associated with ASD (Sivamaruthi et al. 2020) and reduced levels of Phylum Bacteroidota Genus Parabacteroides. Notably, LR supplementation did not prevent the other taxonomic changes in Firmicutes, Bacteroidota (G. Odoribacter), Chlamydiae, Protobacteria, and Bacteroidota (G. Prevotella). The latter is a low-level carbohydrate degrading/fermenting bacteria present in the oral microbiome of children with ASD (Qiao et al. 2018). In combination, these findings indicate that, in contrast to males, P22 females showed much less dysbiosis associated with DE-71. Moreover, LR treatment was effective against their dysbiosis and a great majority of their abnormal developmental, neurobehavioral, and metabolic reprogramming caused by developmental exposure to DE-71. In males LR was effective against 1/3 of male dysbiotic changes at P22. Moreover, LR treatment was less effective against DE-71-induced changes in taxonomic relative abundance at P30.

Despite a drop in fecal LR at P22, perinatal therapy with LR continued to benefit Cohort 1 mice behavior in adulthood including normalizing hyperactivity, anxiety, and exaggerated repetitive activity in females. Indeed, treatment with lysed Lactobacillus can have bioactivity, such as improving affective/cognitive impairments and decreasing CORT levelslr (Varian et al. 2017; Wu et al. 2022). Alternatively, LR may generate biologically active molecules that signal to the brain via the vagus nerve, and influence behavior (Sgritta et al. 2019; Yu et al. 2023). Indeed, acellular postbiotic compounds identified for *L. reuteri* include bioactive histamine, tetrahydrobiopterin, a cofactor for aromatic amino acid hydroxylase enzymes, nitric oxide synthases (Buffington et al. 2021) and bifunctional dihydrofolate synthase/folylpolyglutamate synthase 2 gene-mediated folate metabolism products (Gao Chunxu et al. 2015; Thomas et al. 2016). Other beneficial effects of LR may be indirect by producing antimicrobials that slow the growth of certain harmful bacteria, viruses, yeasts, fungi, and protozoa (Axelsson et al. 1989). Yet other mechanisms underlying the benefits of probiotic treatment range from improved function of host immune system and activation of neuro/endocrine systems, to xenobiotic biotransformation by the gut microbiome (Clarke et al. 2019; Goyal and Saravanan 2023; Mu et al. 2018; Varian et al. 2014).

Although untested, LR may reduce toxicity of PBDEs by increasing their absorption as reported for *Lactobacillus rhamnosus* ability to counteract organophosphate pesticides (Trinder et al. 2016) or other bacteria like *Sphingomonas sp* strain Ss3 that provide dehalogenation (Robrock et al. 2009; Schmidt et al. 1992). Indeed, LR has demonstrated the highest ability among tested probiotics to remove the toxicant bisphenol A from canned beverages (Ju et al. 2019). While microbial-assisted remediation strategies are used to reduce volatile POPs such as polychlorinated biphenyls to 1 ppm in the groundwater (Bala et al. 2022) and PBDEs in sediment (Huang et al. 2019), to our knowledge, no previous studies have explored maternal transfer of probiotics as a potential therapy for toxicant-induced developmental deficits in vivo.

## Conclusions

Collectively, these data support the conclusion that indoor flame retardant chemicals, specifically PBDEs, can act as developmental neurotoxicants and endocrine and metabolic disruptors in exposed males and females. More importantly, our study provides novel evidence that *maternal* probiotic therapy with LR normalizes aberrant postnatal developmental benchmarks and reprogramming of neurological and metabolic homeostasis produced by maternal transfer of PBDEs. The observed benefits included amelioration of abnormal incisor eruption, normalization of male postnatal body weight gain, and normalization of adult female hyperactivity, repetitive behavior, and diabetogenic glucose metabolism. Therefore, the use of gut microbiota-targeted therapies starting before birth may protect against developmental origins of toxicant-related adult disease. Associated LRdependent changes in the gut microbial community may play a role in these phenotypes. By further understafinding the interaction of probiotics and environmental toxicants, particularly their effects on metabolic disruption and neurotoxicity, microbiota-targeted therapies could be leveraged as an early-life intervention via maternal treatment to prevent the influence of an adverse perinatal environment on lifelong disease.

## Supplementary Material

Supplementary Data 2

Supplementary Data 4

Supplementary Data 3

Supplementary Data 1

## Figures and Tables

**Table 1:** Summary of developmental benchmarks, gut microbial community structure and adult physiology and behavior affected by perinatal DE-71 with or without LR supplementation From: Maternal probiotic supplementation protects against PBDE-induced developmental, behavior and metabolic reprogramming in a sexually dimorphic manner: Role of gut microbiome

	DE-71 females		DE-71 males	
	DE-71	DE-71+ LR	DE-71	DE-71 + LR
**POSTNATAL OFFSPRING**				
Body weight	No Δ	No Δ	↓ P26.28	Normalized P28
Eye opening	↑	↑↑	No Δ	No Δ
Incisoreruption	↓ P10.12	↑ P10,12	tP8	↓ P8
Righting reflex	No Δ	No Δ	No Δ	No Δ
**Gut dysbiosis:**				
Alpha diversity P22	↓	Normalized	No Δ	No Δ
Alpha diversity P30	↑	↑	No Δ	↑
Beta diversity P22	No Δ	No Δ	No Δ	Altered
Beta Diversity P30	No Δ	Altered	Altered	Normalized
**ADULT OFFSPRING**				
Marble burying	↑	Normalized	↑	No Δ
OFT AUC	↑	Normalized	No Δ	No Δ
OFT center	↑	Normalized	No Δ	No Δ
GTT	↑ glycemiat = 15	Normalized	↓glycemiat = 30,60	Normalized
GTTAUC	↑ glycemia (p = 0.06)	Normalized	↓	Normalized
lnsulin:Glucose	↑	Normalized	↑ (p = 0.06)	Normalized
Plasma leptin	↓	Normalized	No Δ	No Δ
Plasma IGF-1	No Δ	No Δ	No Δ	No Δ
Plasma T4	No Δ	No Δ	No Δ	No Δ

All outcomes were measured in Cohort 1 except GTT, GTT AUC, Insulin-to-Glucose and plasma leptin which were obtained from Cohort 2 offspring. Arrows indicate statistical significance vs VEH/CON (↑ p < 0.05 and ↑↑ p < 0.01). P, postnatal day; GTT, glucose tolerance test; OFT, Open field test; IGF-1, insulin-like growth factor 1

## Data Availability

The 16S rRNA gene sequences have been deposited in the National Center for Biotechnology Information (NCBI)’s Sequence Read Archive (SRA) under the SRA BioProject (Accession PRJNA1162038).

## Highlights

- Maternal Limosilactobacillus reuteri (LR) supplementation sex-dependently normalized DE-71-induced gut dysbiosis in offspring.
- Maternal DE-71 sex-dependently altered the timing of offspring postnatal developmental benchmarks.
- Maternal LR ameliorated DE-71-induced delay in male body weight gain.
- Maternal LR normalized sex-dependent DE-71-induced reprogramming of adult glucose homeostasis.
- Maternal LR prevented DE-71-induced hyperactivity and repetitive behavior in adult female offspring.

## Abbreviations

16S rRNA: Gene encoding the small subunit ribosomal RNA molecules of ribosomes
ADHD: Attention-deficit/hyperactivity disorder
ANOVA: Analysis of variance
ASD: Autism spectrum disorder
BDE: Brominated diphenyl ether
BW: Body weight
CON: Saline control
CFU: Colony-forming units
Chao1: α-Diversity index
CNS: Central nervous system
Cpt1a: Carnitine palmitoyltransferase 1a
db/db: Diabetic-obese mice with a mutation in the leptin receptor
DE-71: Pentabromodiphenyl ether mixture
DNA: Deoxyribonucleic acid
DOHaD: Developmental origins of adult health and disease hypothesis
ELISA: Enzyme-linked immunosorbent assay
GD: Gestational day
GTT: Glucose tolerance test
HFD: High-fat diet
IGF-1: Insulin-like growth factor 1
KO: Knockout
LB: Luria–Bertani broth
LC–MS: Liquid chromatography–mass spectrometry
LR: *Limosilactobacillus reuteri* (Previously *Lactobacillus reuteri*)
MB: Marble burying
MetS: Metabolic syndrome
MRS: De Man, Rogosa, and Sharpe Media
NDD: Neurodevelopmental disorder
OD_600_: Optical density
OFT: Open field test
ON: Overnight
OTUs: Operational taxonomic units
PBDEs: Polybrominated diphenyl ethers
PCoA: Principal coordinate analysis
PERMANOVA: Permutational multivariate analysis of variance
P: Postnatal day
POPs: Persistent organic pollutants
QMR: Quantitative magnetic resonance
QIIME 2: Rarefaction analysis
qPCR: Quantitative polymerase chain reaction
T2DM: Type II diabetes mellitus
T4: Total thyroxine
VEH/CON: Vehicle control
