# Supplementary material for: Maternal probiotic supplementation protects against PBDE-induced developmental, behavior and metabolic reprogramming in a sexually dimorphic manner: Role of gut microbiome
Source: Arch Toxicol. Author manuscript; Available in PMC 2025 Jun 3. (PMC11748483; doi:10.1007/s00204-024-03882-4)
Supplement: Supplementary Data 1 [file NIHMS2043947-supplement-Supplementary_Data_1.pdf]

Maternal Probiotic Supplementation Protects Against PBDE-Induced Developmental, Behavior and Metabolic Reprogramming in a Sexually Dimorphic Manner: Role of Gut Microbiome

Maximillian E. Denys[1,†], Elena V. Kozlova[1,2,†], Rui Liu[3], Anthony E. Bishay[1], Elyza A. Do[3,4], Varadh Piamthai[3], Yash V. Korde[1], Crystal N. Luna[1], Artha A. Lam[1], Ansel Hsiao[3] and Margarita C. Curras-Collazo[1,*]

†Maximilian E. Denys and Elena V. Kozlova have contributed equally to this work.

Orcid IDs: M.E.D.: 0000-0002-7423-6265; E.V.K.: 0000-0002-4691-6618; A.E.B.: 0000-0002-6057-3770; E.A.D.: 0000-0003-3264-8138; V.P.: 0009-0008-8273-8653; C.N.L.: 0009-0003-3003-0665; A.L.: 0009-0001-1023-5204; M.C.C.: 0000-0002-0189-4179

[1]Department of Molecular Cell and Systems, University of California, Riverside, CA, USA
[2]Neuroscience Graduate Program, University of California, Riverside, CA, USA
[3]Department of Microbiology and Plant Pathology, University of California, Riverside, CA, USA
[4]Division of Biomedical Sciences, School of Medicine, University of California Riverside, Riverside, CA, USA

***Address correspondence to:**
Dr. Margarita C. Curras-Collazo, Ph.D
Professor of Neuroscience
Department Molecular, Cell and Systems Biology
University of California, Riverside
Riverside, CA 92521
951-827-3960
mcur@ucr.edu

Declarations

Disclaimer
Research reported in this publication was supported by the National Institute of Environmental Health Sciences of the National Institutes of Health under Award Numbers F31ES034304 and F31AI179030. The content is solely the responsibility of the authors and does not necessarily represent the official views of the National Institutes of Health.

Funding
This work was supported by a University of California Chancellor's Undergraduate Research Fellowship (M.E.D.); a Danone North America Gut Microbiome, Yogurt and Probiotics Fellowship Grant and a University of California Office of the President UC-Hispanic Serving Institutions Doctoral Diversity Initiative, President's Pre-Professoriate Fellowship (UC-HSI DDI) and F31ES034304 (E.V.K.); NIH/NIGMS R35GM124724 (A.H.); F31AI179030 (E.A.D.) and UCR Academic Senate grant (M.C.C.).

This work was supported by National Institute of Health grant number F31ES034304

Acknowledgements

Conflicts of interests/Competing interests
The authors report no conflicts of interests and have no competing interests to declare.

Ethics approval
Care and treatment of animals was performed in accordance with guidelines from and approved by the University of California, Riverside Institutional Animal Care and Use Committee (AUP#5, 20210031).

Consent to participate
Not applicable.

Consent for publication
All authors reviewed and approved the final manuscript.

Data availability
The 16S rRNA gene sequences have been deposited in the National Center for Biotechnology Information (NCBI)'s Sequence Read Archive (SRA) under the SRA BioProject Accession PRJNA1162038

Not applicable.

Code Availability
Not applicable.

CRediT authorship contribution statement
Conceptualization, M.E.D., E.V.K., A.H., M.C.C.; **Methodology**, M.E.D., E.V.K., A.H., M.C.C.; **Validation**, M.E.D., E.V.K., M.C.C.; **Formal Analysis**, M.E.D., E.V.K., A.E.B., R.L., E.A.D., M.C.C.; **Investigation**, M.E.D., E.V.K., A.E.B., E.A.D., V.P., Y.V.K., C.N.L., A.A.L., M.C.C.; **Writing – Original Draft**, M.E.D., E.V.K., A.E.B., M.C.C.; **Writing – Reviewing and Editing**, M.E.D., E.V.K., A.E.B., A.H., M.C.C.; **Visualization**, M.E.D., E.V.K., A.E.B., R.L., M.C.C.; **Resources**, A.H., M.C.C.; **Data Curation**, M.E.D., E.V.K., A.E.B., R.L., M.C.C.; **Supervision**, E.V.K., A.H., M.C.C.; **Project Administration**, E.V.K., A.E.B., M.C.C.; **Funding Acquisition**, E.V.K., A.H., M.C.C. All authors reviewed and approved the manuscript.

Supplementary Figure 1

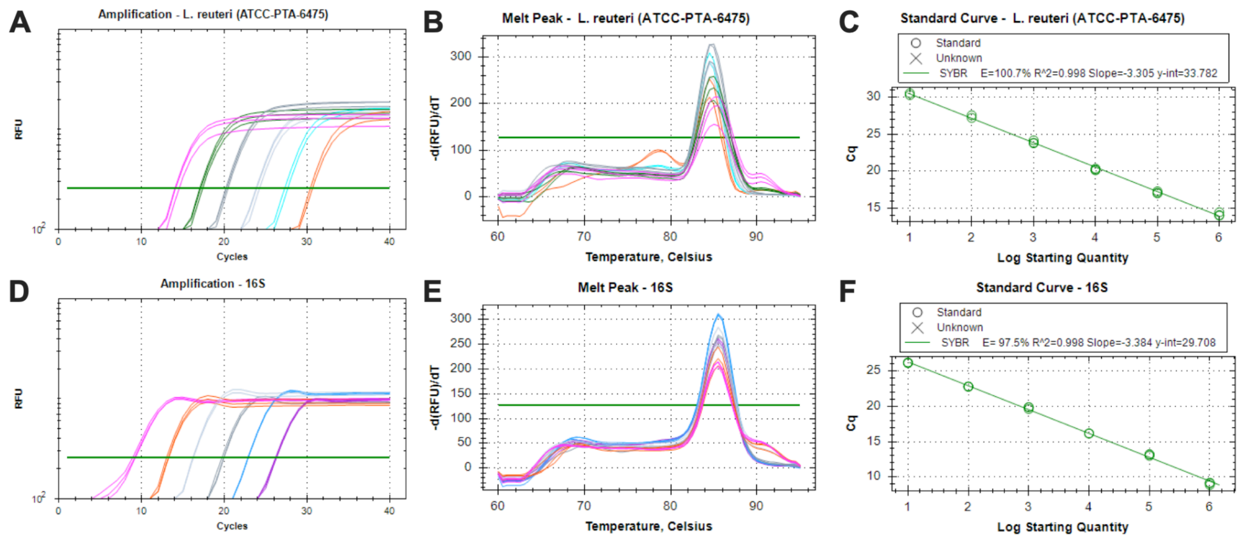

Supplementary Figure 1 (Related to Figure 1). (A) RT-qPCR amplification curve for *L. reuteri* (ATCC-PTA-6475) primer standard curve. **(B)** corresponding melt peak, indicated on target amplification. **(C)** Corresponding standard curve of 10-fold DNA template dilutions indicating 100.7% efficiency. **(D)** RT-qPCR amplification curve for 16S bacteria primer standard curve. **(E)** corresponding melt peak, indicated on target amplification. **(F)** Corresponding standard curve of 10-fold DNA template dilutions indicating 97.5% efficiency.

Supplementary Figure 2

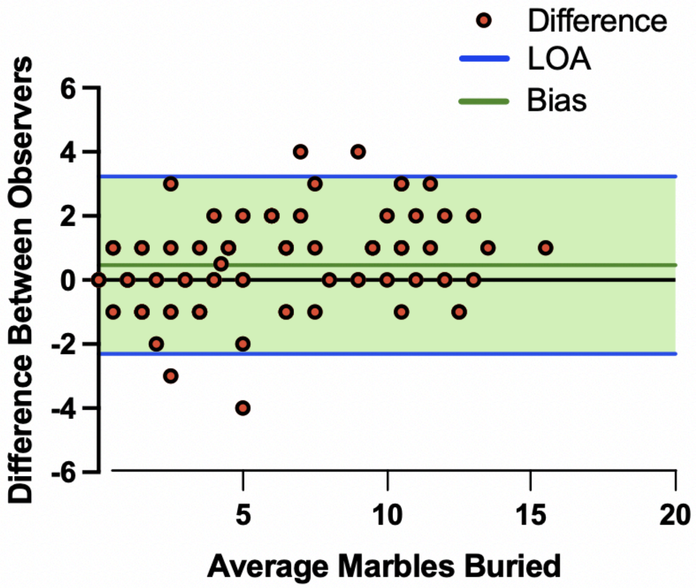

Supplementary Figure 2. Bland-Altman plot for marble burying scores (Related to Figure 6). Bland-Altman bias plot (mean±s.d.) was used to test the validity and reproducibility between two independent observers blind to exposure group. Analysis revealed a very small mean of the differences between observer scores (Bias, 0.46±1.41) and a precision measured as limits of agreement (LOA), average difference ± 1.96 standard deviation of the difference of -2.31- 3.23, indicating negligible skewing by either observer.

Supplementary Figure 3

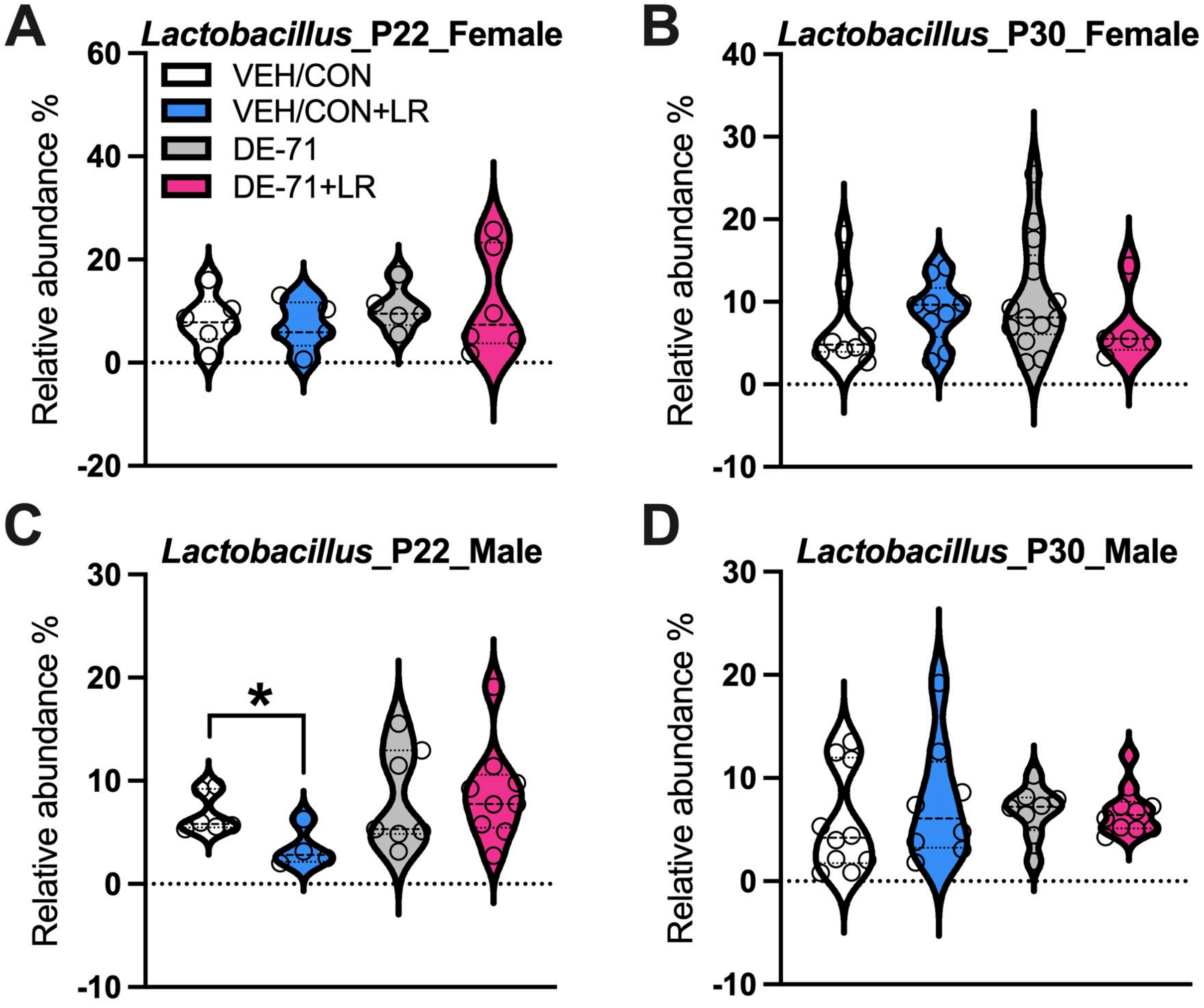

Supplementary Figure 3. (Related to Figures 3 and 4). (**B-E**) Relative abundance of genus *Lactobacillus* in **(A)** P22 females, **(B)** P30 females, **(C)** P22 males, **(D)** P30 males. PERMANOVA results indicated the only group difference was in P22 males VEH/CON vs VEH/CON+LR. *statistical difference vs VEH/CON (*p<0.05). n=6-10/group.

Supplementary Figure 4

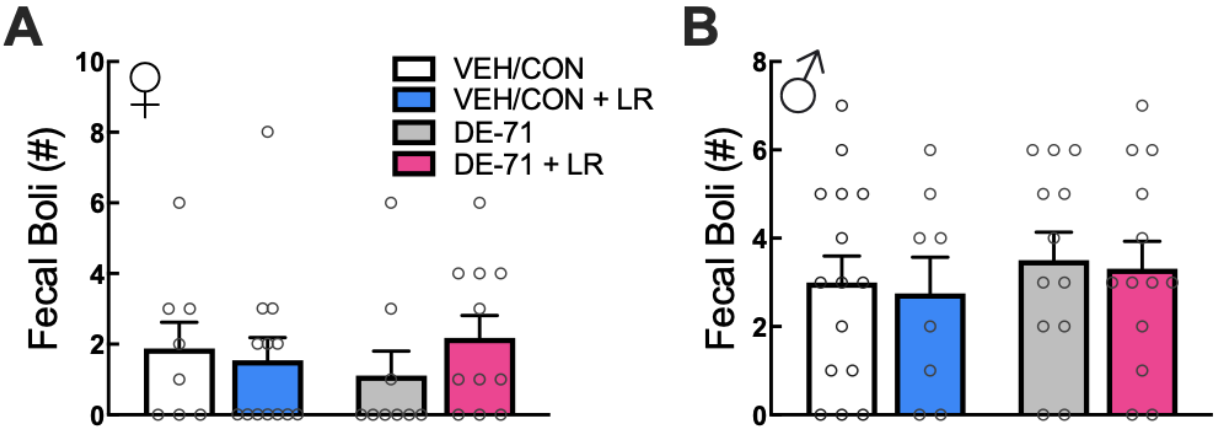

Supplementary Figure 4. Number of fecal boli deposited in the arena during the open field test (Related to Figure 6). Mice were tested in the open field test for 10 min. (**A**) female offspring. (**B**) male offspring. *n*, 8-15/group. Values represent mean ± SEM. Two-Way ANOVA results indicated no group differences in exposure or treatment.

Supplementary Figure 5

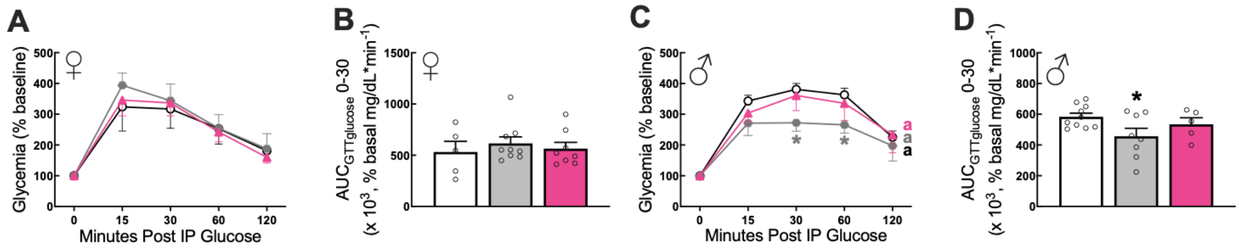

Supplementary Figure 5 (Related to Figure 7). (**A,C**) Glycemia expressed as % basal. (**B,D**) Mean values for the integrated area under the GTT % basal glucose curve ($AUC_{IPGTTglucose}$) from 0-30 min. Values represent mean±s.e.m. *indicates statistical difference vs VEH/CON, *p<0.05; [a]significant increase from within group baseline (p<0.05-0.0001). n, 5-12/group.

Supplementary Figure 5 Statistics. (**A**) Glycemia percent basal females. (Two-way RM ANOVA: *Treatment* effect $F_{(2,19)} = 0.14$, $p = 0.87$; *Time* effect $F_{(4,76)} = 34.65$, $p < 0.0001$; *Treatment* × *Time* $F_{(8,76)} = 0.27$, $p = 0.97$). (**B**) corresponding $AUC_{GTTglucose}$, (One-way ANOVA: *Treatment effect* $F_{(2,19)} = 0.33$, $p = 0.7207$). (**C**) Glycemia percent basal males. (Two-way RM ANOVA: *Treatment* effect $F_{(2,20)} = 2.50$, $p = 0.107$; *Time* effect $F_{(4,80)} = 54.61$, $p < 0.0001$; *Treatment* × *Time* $F_{(8,80)} = 1.36$, $p = 0.23$). Holm Sidak's *post hoc* test for multiple comparisons showed a significant decrease in glycemia in DE-71, but not DE-71+LR vs VEH/CON at t=30 and t=60 (p<0.05). (**D**) The corresponding $AUC_{GTTglucose}$ showed reduced glycemia over the first 30 min in DE-71 only (Males, One-way ANOVA: *Exposure effect* $F_{(2,20)} = 3.06$, $p = 0.07$). Holm Sidak's *post hoc* test showed a significantly lower $AUC_{glucose}$ in DE-71, but not DE-71 + LR, vs VEH/CON (p<0.05).

Supplementary Table 1. Effect of DE-71 and LR supplementation on fasting glycemia, body weight, body composition and plasma leptin in offspring

	VEH/CON	DE-71	DE-71+LR
**FEMALES**			
Fasting Glycemia 9h (mg/dL)	131.3 ± 5.40	144.0 ± 5.41	118.0 ± 8.90^
Fasting Glycemia 11h (mg/dL)	97.9 ± 7.08	111.0 ± 7.15	112.9 ± 9.50
Fed Body Weight (g)	21.8 ± 0.439	20.8 ± 0.359	21.6 ± 0.393
Fat Mass (% Body Weight)	16.2 ± 1.92	16.6 ± 0.956	16.8 ± 1.32
Lean Mass (% Body Weight)	89.5 ± 0.658	88.0 ± 0.581	87.9 ± 0.936
Fed Plasma Leptin (% VEH/CON)	100 ± 8.95	73.6 ± 2.01*	92.2 ± 7.82
**MALES**			
Fasting Glycemia 9h (mg/dL)	123.0 ± 4.13	112.9 ± 3.0	110.9 ± 4.6
Fasting Glycemia 11h (mg/dL)	122.4 ± 4.16	136.5 ± 6.32	125.4 ± 6.81
Fed Body Weight (g)	27.8 ± 0.340	29.2 ± 1.10	26.3 ±1.48
Fat Mass (% Body Weight)	21.5 ± 1.56	23.2 ± 1.78	20.2 ± 2.27
Lean Mass (% Body Weight)	84.9 ± 1.10	82.9 ± 1.39	86.0 ± 1.36
Fed Plasma Leptin (% VEH/CON)	100 ± 12.5	134 ± 20.5	97.4 ± 6.57

Data was acquired from Cohort 2 offspring at PND 150-180. Data are expressed as mean±s.e.m.
* statistically different from VEH/CON; * p<0.05
^ statistically different from DE-71; ^ p<0.05

Supplementary Table 2 Statistics.

Glycemia 9h ON females. (One-way ANOVA: *Treatment effect* $F_{(2,19)}=3.928$, $p=0.04$).

Glycemia 11h ON females (One-way ANOVA: *Treatment effect* $F_{(2,23)}$=1.00, p=0.384).
Tukey's *post hoc* test revealed lower fasting glycemia in DE-71+LR vs DE-71 after 9 hr ON fast (p<0.05).

Glycemia 9h ON males. (One-way ANOVA: *Treatment effect* $F_{(2,22)}$=2.32, p=0.12)

Glycemia 11h ON males (One-way ANOVA: *Treatment effect* $F_{(2,20)}$=1.975, p=0.1649).

Body weight females. (One-way ANOVA: *Treatment effect* $F_{(2,23)}$=1.727, p=0.200).

Body weight males. (One-way ANOVA: *Treatment effect* $F_{(2,20)}$=2.103, p=0.1483).

Fat mass females (*Treatment effect* $F_{(2,22)}$=0.9940, p=0.386.

Fat mass males (One-way ANOVA: *Treatment effect* $F_{(2,20)}$=1.371, p=0.277). Likewise, there were no group differences in lean mass in females or males.

Fed state leptin females. (Welch's ANOVA: *Treatment effect* $F_{(3,9.27)}$=7.008, p<0.01). Dunnet's T3 *post hoc* test indicated significantly reduced leptin levels in DE-71 females compared to VEH/CON females (p<0.05).

Fed state leptin males. (Males, One-Way ANOVA: *Treatment effect* $F_{(2,15)}$=1.963, p=0.175).

Supplementary Figure 6

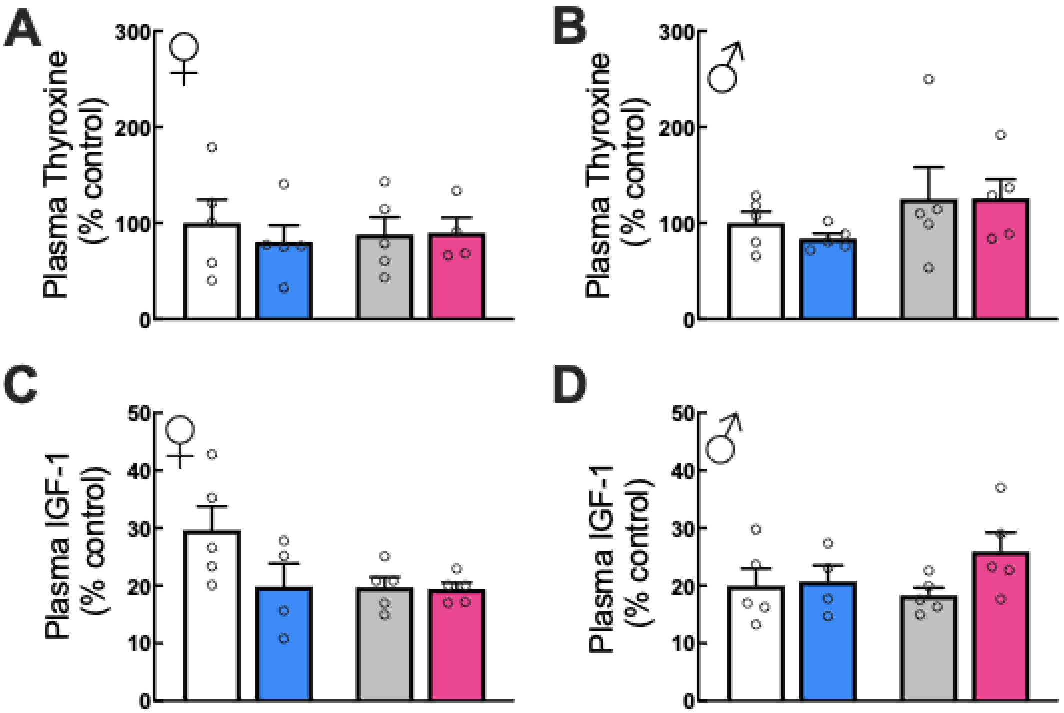

Supplementary Figure 6. (Related to Table 1). Effects of DE-71 and LR supplementation on offspring T4 and IGF-1 levels (A-B) Plasma T4 levels were measured in adulthood with ELISA and normalized to percent VEH/CON. **(C-D)** Plasma IGF-1 levels in adult offspring. *n*, 6-10 dams/group. Values represent mean ± SEM. Two-Way ANOVA inidcated no significant effect of exposure or treatment.

Supplementary Statistical Information

Figure 1. Gut colonization of L. reuteri in dams and their offspring after maternal treatment. (B) Simple linear regression: R^2=0.4705, F(1,8)=7.107, *p*=.029. Pearson's Correlation: r=0.6859, R^2=0.4705, *p*=0.0143. (**C**) (RM One-way Mixed Effects model: *Treatment effect* $F_{(2, 10)}$=9.341, *p*<0.01, Tukey's *post-hoc*: 11 doses vs basal, *p*=0.917; 25 doses vs basal (*p*=0.009); 25 vs 11 doses (*p*=0.016). (**D**) (One-Way ANOVA: *Treatment effect* $F_{(2, 13)}$=20.76, *p*<0.0001, Tukey's *post-hoc*: LR PND 22 vs CON PND 22 (*p*<0.001); LR PND 22 vs LR PND 30 (*p*=0.0004); LR PND 30 VS CON PND22, *p*=0.9930).

Figure 5. Maternal LR treatment protects against DE-71-induced developmental deficits. (**A**) Body weight females. RM Mixed Effects Model: *Treatment effect* $F_{(3, 40)}$=2.542, *p*=0.069); *Time effect* $F_{(1.843, 73.19)}$=1766, *p*<0.0001; *Treatment* x *Time* $F_{(42, 556)}$=1.572, *p*=0.014). Holm Sidak's *post hoc* test for multiple comparisons showed that maternal LR treatment significantly increased body weight in female DE-71+LR vs DE-71 on PND 4, 6, 8, 10 and 20 (*p*<0.05-0.01) and VEH/CON+LR vs VEH/CON on day 4, 6 (*p*<0.05). (**B**) Body weight males. RM Mixed Effects Model: *Treatment effect* $F_{(3, 51)}$=2.080, *p*=0.115; *Time effect* $F_{(14, 705)}$=2081, *p*<0.0001; *Treatment* x *Time* $F_{(42, 705)}$=3.708, *p*<0.0001). Holm Sidak's *post hoc* test for multiple comparisons showed a significant *decrease* in body weight in DE-71 but not DE-71+LR vs VEH/CON on day 26, 28 (*p*<0.05-0.01). Mean body weight was also significantly elevated in DE-71+LR vs DE-71 on PND 28 (*p*<0.05). Mean body weight was lower in VEH/CON+LR vs VEH/CON on PND 24-30 (*p*<0.01-0.0001). (**C**) Body length females. (RM Mixed Effects Model: *Treatment effect* $F_{(3, 39)}$=1.557, *p*=0.2152; *Time effect* $F_{(6, 230)}$=1950, *p*<0.0001; *Treatment x Time* $F_{(18,230)}$=1.876, *p*=0.02) Holm Sidak's *post hoc* test showed that LR treatment increased body length in DE-71+LR vs DE-71 in females on PND 10 (*p*<0.05) as well as in DE-71+LR vs VEH/CON+LR on PND 14 (*p*<0.01). (**D**) Body length males (RM Mixed Effects Model: *Treatment effect* $F_{(3, 51)}$=1.613, *p*=0.1979; *Time effect* $F_{(4.225, 211.3)}$=2733, *p*<0.0001; *Treatment x Time* $F_{(18, 300)}$=1.236, *p*=0.2305). In males, mean body length was greater in DE-71+LR vs VEH/CON+LR on PND 8 (*p*<0.01). (**E**) Tail length females. (RM Two-Way Mixed Effects Model: *Treatment effect* $F_{(3, 39)}$=3.771, *p*=0.02; *Time effect* $F_{(1.651, 63.30)}$=668.7, *p*<0.0001; *Treatment* x *Time* $F_{(18, 230)}$=0.5088, *p*=0.9527). Holm Sidak's *post hoc* test for multiple comparisons showed that combined treatment with DE-71+LR increased mean tail length vs VEH/CON on PND 2, 6, 8, 12, 14 (*p*<0.05-0.01) and vs DE-71 on PND 8 (*p*<0.05). LR treatment (VEH/CON+LR) increased mean tail length vs VEH/CON on PND 2, 6, 8. **(F)** Tail length males (RM Mixed Effects Model: *Treatment effect* $F_{(5, 31)}$=1.331, *p*=0.0181; *Time effect* $F_{(6, 300)}$=1345, *p*<0.0001; *Treatment* x *Time* $F_{(18, 300)}$=1.7518, *p*<0.031. In males, mean tail length was significantly greater in DE-71+LR vs DE-71 on PND 10 (*p*<0.05) and vs VEH/CON+LR on PND 10, 12, 14 (*p*<0.05). **(G)** righting reflex in females. (Females, RM Two-Way Mixed Effects Model: *Treatment effect* $F_{(3, 39)}$=0.925, *p*=0.4379; *Time effect* $F_{(4, 148)}$=30.96, *p*<0.0001; *Treatment x Time* $F_{(12,)}$=1.048, *p*=0.4084). Holm Sidak's *post hoc* test for multiple comparisons showed a significant improvement in DE-71+LR vs DE-71 on PND 4 (*p*<0.05) and vs VEH/CON on PND 10 (*p*<.05). (**H**) Righting reflex males. (Males, RM Two-Way Mixed Effects Model: *Treatment effect* $F_{(3,51)}$=1.189, *p*=0.3231; *Time effect* $F_{(4, 196)}$=39.76, *p*<0.0001; *Treatment x Time* $F_{(12,196)}$=1.734, *p*=0.0620). In males, reduced mean latency scores were seen in DE-71+LR vs DE-71 at PND 6 (*p*<0.05) and vs VEH/CON+LR on PND 2 (*p*<0.05). (**I**) Eye opening females. (Females, RM Two-Way ANOVA: *Treatment effect* $F_{(3, 40)}$=3.697, *p*<0.05; *Time effect*

$F_{(2,80)}$=35.75, $p<0.0001$; *Treatment x Time* $F_{(6, 80)}$=2.262, $p<0.05$). Holm Sidak's *post hoc* test for multiple comparisons showed significantly sooner eye opening at PND 14 in DE-71+LR, DE-71, VEH/CON+LR vs. the VEH/CON group, which contained the most delayed phenotype (p<0.05-0.001). (**J**) Eye opening males. (Males, RM Two-Way ANOVA: *Treatment effect* $F_{(3, 47)}$=0.141, p=0.9351; *Time effect* $F_{(2, 94)}$=76.77, $p<0.0001$; *Treatment x Time* $F_{(6,)}$=2.42, $p<0.05$). Holm Sidak's *post hoc* test revealed expedited eye opening in VEH/CON+LR vs DE-71 on PND 14 ($p<0.05$). (**K**) Incisor eruption females. (Females, RM Two-Way ANOVA: *Treatment effect* $F_{(3,40)}$=4.786, $p<0.01$; *Time effect* $F_{(4, 160)}$=159.6, $p<0.0001$; *Treatment x Time* $F_{(12, 160)}$= 5.23, $p<0.0001$). Holm Sidak's *post hoc* test for multiple comparisons showed a significant delay in incisor eruption in DE-71 vs VEH/CON on PND 10 and 12 (p<0.01-0.0001) and in VEH/CON+LR on PND 12 ($p<0.001$) but DE-71+LR showed improved mean score vs DE-71 on PND 10 and 12 ($p<0.001$). (**L**) Incisor eruption male. (Males, RM Two-Way ANOVA: *Treatment effect* $F_{(3, 47)}$=0.977, p=0.4116; *Time effect* $F_{(2,192)}$=213.4, $p<0.0001$; *Treatment x Time* $F_{(12,192)}$=2.28, $p<0.05$. Holm Sidak's *post hoc* test for multiple comparisons showed a significantly better mean incisor eruption scores in DE-71 vs VEH/CON on PND 8 ($p<0.05$). This was normalized in DE-71+LR vs DE-71 ($p<0.05$).

Figure 6. Maternal LR treatment prevents repetitive behavior in DE-71-exposed female but not male offspring. (**A**) Marble burying females. (Two-way ANOVA *Exposure* effect ($F_{(1,37)} = 16.75$, $p<0.001$); *Treatment* effect ($F_{(1,37)} = 9.849$, $p<0.01$); *Exposure* × *Treatment* ($F_{(1,37)}$ =0.4842, p=0.4909). Holm Sidak's *post hoc* test showed significantly increased marble burying in DE-71 vs. both VEH/CON ($p<0.01$) and vs. DE-71+LR ($p<0.05$). (**B**) Male marble burying. (Two-way ANOVA *Exposure* effect ($F_{(1,43)} = 25.38$, $p<0.0001$); *Treatment* effect ($F_{(1,43)} = 2.36$, p=0.1317); *Exposure* × *Treatmen*t ($F_{(1,43)} = 0.521$, p=0.4741). Holm Sidak's *post hoc* test for multiple comparisons showed exaggerated marble burying in DE-71 ($p<0.01$) and DE-71+LR vs VEH/CON ($p<0.01$). (**C**) open field arena females (RM Two-Way ANOVA: *Treatment* effect ($F_{(3,35)} = 19.00$, $p<0.0001$); *Time* effect ($F_{(9,315)} = 5.979$, $p<0.0001$); *Exposure* × *Time* ($F_{(27,315)} = 1.616$, $p<0.05$). Holm Sidak's *post hoc* test for multiple comparisons showed a significant increase in distance traveled in DE-71 vs VEH/CON at t=1,2,6-10 min (p<0.05-0.01). Female DE-71+LR traveled less distance vs DE-71 at all time points (p<0.01-0.0001), suggesting that maternal LR ameliorated the hyperactive effects of DE-71. VEH/CON+LR also traveled less distance vs VEH/CON at t=1,2,3,4,7,9 min (p<0.05-0.0001), indicating an effect of LR to decrease locomotion. (**D**) Open field arena males. (RM Two Way ANOVA: *Treatment* effect $F_{(3,44)} = 1.803$, p=0.1605; *Time* effect $F_{(1.624, 71.44)} = 4.852$, $p<0.05$; *Treatment* × *Time* $F_{(27,396)} = 0.4962$, p=0.9850). Holm Sidak's *post hoc* test for multiple comparisons showed no effect of DE-71 on distance traveled However, LR treatment reduced distance traveled in VEH/CON at t=3,5,7 ($p<0.05$). (**E**) Cumulative distance traveled females. (Two-way ANOVA, *Exposure* effect $F_{(1,35)} = 12.35$, p=0.0112; *Treatment* effect $F_{(1, 35)}$ =44.97, $p<0.0001$; *Exposure x Treatment* $F_{(1,35)} = 2.332$, p=0.1358). Holm Sidak's post hoc revealed that LR treatment produced significantly less cumulative distance traveled in VEH/CON+LR ($p<0.01$) and DE-71+LR ($p<0.0001$) vs corresponding controls. (**F**) Cumulative distance traveled males. (Two-way ANOVA, *Exposure* effect $F_{(1,44)} = 1.913$, p=0.1736; *Treatment* effect $F_{(1,44)}$ =2.257, p=0.1402; *Exposure x Treatment* $F_{(1,44)} = 2.863$, p=0.0977). Fisher's LSD revealed that LR treatment produced significantly less cumulative distance traveled in VEH/CON+LR ($p<0.05$) and less travel in VEH/CON+LR vs DE-71+LR ($p<0.05$). (**I**) Distance traveled by zone females. (Two-way ANOVA, *Exposure* effect ($F_{(1,37)} = 0.3008$, p=0.5767); *Treatment* effect

($F_{(1,37)}$ =1.202, p<0.280); *Exposure x Treatment* ($F_{(1, 37)} = 6.828$, p<0.013). Holm Sidak's *post hoc* test indicated greater travel in DE-71 vs VEH/CON females (p<0.05). LR supplementation of DE-71 females normalized travel time in center vs DE-71 (p<0.05). (**J**) Distance traveled by zone males. (Two-way ANOVA, *Exposure* effect ($F_{(1,44)} = 0.214$, p=0.646); *Treatment* effect ($F_{(1,44)} = 1.486$, p=0.2294); *Exposure x Treatment* ($F_{(1,44)} = 1.961$, p=0.167). Holm Sidak's *post hoc* test indicated no group differences.

Figure 7. Maternal LR treatment reduced DE-71 effects on glucose intolerance and insulin-to-glucose ratio in a sex-dependent manner. (**A**) Glycemia females. (Two-way RM ANOVA: *Treatment* effect $F_{(2,19)} = 1.1$, p =0.37; *Time* effect $F_{(4,76)} = 52.08$, p<0.0001; *Treatment* × *Time* $F_{(8,76)} = 0.94$, p =0.49). Holm Sidak's *post hoc* test for multiple comparisons showed that except for DE-71 females, glycemia was significantly greater at 15-60 min post-glucose injection and returned to normal by 120 min when compared to baseline (p<0.05) DE-71 but not DE-71 + LR females displayed greater glycemia (p<0.05) vs VEH/CON at t=15 (p<0.05). (**B)** $AUC_{GTTglucose}$, females. (One-way ANOVA: *Treatment* effect $F_{(2,19)}$=2.102, p=0.15, Holm Sidak's *post hoc* test DE-71 vs VEH/CON p=0.06). When glycemia was represented as a percent of basal, there were no group differences in magnitude at any timepoint (Two-way RM ANOVA: *Treatment* effect $F_{(2,19)} = 0.14$, p =0.87; *Time* effect $F_{(4,76)} = 34.65$, p<0.0001; *Treatment* × *Time* $F_{(8,76)} = 0.27$, p=0.97) or in corresponding $AUC_{GTTglucose}$, (One-way ANOVA: *Treatment effect* $F_{(2,19)}$=0.33, p=0.7207). (**C**) Plasma insulin females. (RM Two-way ANOVA, *Treatment* effect ($F_{(2,19)} = 2.03$, p=0.15); *Time* effect ($F_{(2,38)} = 15.71$, p<0.0001); *Treatment* × *Time* ($F_{(4,38)} = 1.45$, p =0.24). Plasma insulin increased at t=15 and/or t=30 vs baseline (Holm Sidak's *post hoc* test p<0.05) Fisher's LSD *post hoc* test showed significantly elevated insulin at t=15 for DE-71 vs VEH/CON and vs DE-71 + LR (p<0.05). (**D**) Insulin to glucose ratio females. (RM Two-way ANOVA, *Treatment* effect ($F_{(2,17)} = 5.16$, p =0.02); *Time* effect ($F_{(2,34)}$ =5.50, p<0.01); *Treatment* × *Time* ($F_{(4,34)} = 2.16$, p=0.095). Holm Sidak's *post hoc* test revealed that DE-71 females had a significantly higher ratio vs VEH/CON at t=15 (p<0.05) and vs DE-71+LR at t=15 (p<0.05) and t=30 min (p<0.01). Moreover, only DE-71 females showed an elevated ratio at t=15 and t=30 (p<0.01). (**E**) Glycemia males. (Two-way RM ANOVA: *Treatment* effect $F_{(2,20)} = 1.84$, p =0.18; *Time* effect $F_{(2.51,50.34)} = 66.32$, p<0.0001; *Treatment* × *Time* $F_{(8,80)} = 1.163$, p =0.332). Holm Sidak's *post hoc* test for multiple comparisons showed a significant *decrease* in glycemia post-glucose challenge in DE-71, but not DE-71 + LR, vs VEH/CON at t=30 (p<0.01) and t=60 (p<0.05). (**F**) $AUC_{GTTglucose}$ males. (One-way ANOVA: *Treatment effect* $F_{(2,20)}$=2.350, p=0.12, Holm Sidak's *post hoc* test p<0.05). (**G**) Insulin males. (RM Two-way ANOVA, *Treatment* effect ($F_{(2,14)} = 0.305$, p=0.742); *Time* effect ($F_{(2,28)} = 12.06$, p<0.001); *Treatment* × *Time* ($F_{(4,28)} = 0.157$, p=0.957). Holm Sidak's *post hoc* test indicated an apparent (VEH/CON, p=0.07) or a significant insulin response (DE-71, DE-71+LR, p<0.01-0.05) at t=15 and t=30. (**H**) Insulin to glucose ratio males. (Two-way RM ANOVA, *Treatment* effect ($F_{(2,14)} = 1.25$, p=0.316); *Time* effect ($F_{(2,28)} = 1.57$, p=0.226); *Treatment* × *Time* ($F_{(4,28)} = 0.73$, p=0.582).
